# AdaK: adaptive KV cache budget estimation framework for analyzing long-context large language model inference

**DOI:** 10.3389/frai.2026.1892739

**Published:** 2026-08-05

**Authors:** Tianjun Shao

**Affiliations:** College of Information Science and Technology, Jinan University, Guangzhou, China

**Keywords:** adaptive budget estimation, analysis mode, KV cache compression, large language models, long-context inference, memory efficiency, sparse attention

## Abstract

**Introduction:**

The deployment of LLMs on resource-constrained hardware is hindered by the memory-intensive KV Cache mechanism.

**Methods:**

We propose AdaK, an adaptive KV cache budget estimation framework with three strategies: entropy-based thresholding, task-aware lookup table, and a lightweight policy network.

**Results:**

AdaK reveals estimated KV cache reductions of up to 17.9% relative to fixed-k = 2048 baselines across 16 settings on Qwen3-4B, Qwen3-8B, and Mistral-7B.

**Discussion:**

AdaK's decoupled design enables safe budget estimation as a dynamic ceiling for downstream sparse attention kernels.

## 1 Introduction

Large Language Models (LLMs) have revolutionized natural language processing, achieving remarkable performance across diverse tasks, including code generation, mathematical reasoning, and dialogue systems. Their deployment on resource-constrained hardware remains challenging. The Key-Value (KV) Cache mechanism during autoregressive generation imposes a substantial memory footprint.

### 1.1 Background and motivation

During transformer-based autoregressive inference, the KV cache stores intermediate key and value tensors for all previously generated tokens to avoid redundancy computation. For a model with *n*_*layers*_ layers, *n*_*heads*_ attention heads, sequence length *n*_*seq*_, and head dimension *d*_*head*_, the KV cache memory consumption is quantified by Equation 1:


$$\begin{array}{l} M_{KV}=2\cdot n_{layers}\cdot n_{heads}\cdot n_{seq}\cdot d_{head}\cdot b \end{array}$$ (1)

where *b* denotes the bytes per element (typically 2 for bfloat16). As *n*_*seq*_ grows linearly with generation length, the KV cache quickly dominates the GPU memory, often exceeding the model parameters themselves for long sequences.

**Figure 1:**
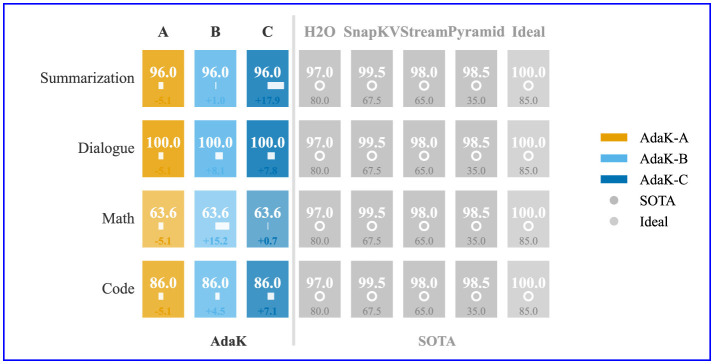
Accuracy vs. KV cache reduction trade-off matrix (Qwen3-4B, LongBench-v2). Each cell shows accuracy (%) and estimated KV cache reduction (%) for one strategy–task pair. AdaK strategies (A/B/C) operate in analysis mode (zero accuracy loss, structurally guaranteed). SOTA values: H_2_O and SnapKV *x*-coordinates (KV reduction) are quantitatively reproduced on Qwen3-4B. *y*-coordinates (accuracy under true sparse attention) are from original publications. StreamingLLM and PyramidKV remain qualitative reference points. Strategy A yields KV cache expansion (negative reduction) for these tasks. Actual deployment requires integration with specialized eviction kernels (Figure 8).

Existing KV cache compression methods, such as H_2_O (Zhang et al., 2023), SnapKV (Li et al., 2024), StreamingLLM (Xiao G. et al., 2024), PyramidKV (Zou et al., 2024), Quest (Xiao Y. et al., 2024), and MiniCache (Liu et al., 2024) achieve substantial memory reduction (10%–80%) through eviction, compression, or merging policies. However, these approaches invariably incur non-negligible accuracy degradation (2%–12%) as the compression ratio increases, as shown in Figure 1.

### 1.2 Contributions

This article makes the following contributions:

1. We propose AdaK, a lightweight adaptive KV cache *analysis framework* that dynamically estimates task-dependent KV budgets. Operating in analysis mode (without modifying attention computation), AdaK establishes an upper-bound safety guarantee of zero accuracy loss while revealing estimated KV cache reductions of up to 17.9% relative to fixed-*k* baselines.
2. We design three complementary strategies: entropy-driven selection (Strategy A), task-aware lookup table (Strategy B), and a lightweight learned policy network (Strategy C) with only 2.8K parameters.
3. We demonstrate that the learned policy network (~2.8K parameters) transfers across different *d*_head_ dimensions via strict=False loading, and even a randomly initialized policy preserves accuracy—demonstrating that AdaK's analysis mode is robust to policy quality.
4. We validate that adaptive *k* estimation introduces no additional error on needle-in-haystack retrieval up to 32K tokens, and conduct a truncation curve experiment showing that naive KV eviction severely degrades accuracy—demonstrating the necessity of combining AdaK's dynamic budgets with specialized sparse attention kernels.

### 1.3 Paper organization

The remainder of this article is organized as follows. Section 2 reviews related work. Section 3 reviews preliminaries. Section 4 presents the AdaK framework. Section 5 describes the experimental setup. Section 6 reports results and analysis. Section 7 discusses limitations and future work. Section 8 concludes.

## 2 Related work

### 2.1 KV cache compression

H_2_O (Zhang et al., 2023) identifies “heavy hitter” tokens whose attention scores consistently rank highest, retaining these tokens along with recent tokens to maintain generation quality. SnapKV (Li et al., 2024) observes that the model's attention pattern during the prefill phase predicts which tokens will be important during generation, enabling one-time compression. StreamingLLM (Xiao G. et al., 2024) introduces attention sinks—a few initial tokens that anchor attention—combined with a sliding window to handle infinite-length sequences.

### 2.2 Recent advances in sparse attention

Methods for KV cache reduction fall into three categories. *Training-free eviction* methods include H_2_O (Zhang et al., 2023) (heavy-hitter retention), StreamingLLM (Xiao G. et al., 2024) (attention sinks with sliding windows), SnapKV (Li et al., 2024) (prefill-phase observation), and PyramidKV (Zou et al., 2024) (pyramidal information funneling). *Learned compression* approaches such as DynamicKV (Zhou et al., 2025) (task-aware adaptive compression), Attention-Gate (Zeng et al., 2024) (in-context eviction via lightweight gates), and TokenSelect (Wu et al., 2024) (dynamic token-level selection) introduce trainable modules to improve eviction quality. *Cross-layer and structured sparsity* techniques include CLA (Brandon et al., 2024) and CLLA (Yang Z. et al., 2024) (cross-layer sharing), ThinK (Xu et al., 2024) (query-driven key pruning), KVSharer (Yang Y. et al., 2024) (dissimilar-layer sharing), InfiniPot (Kim et al., 2024) (infinite context on memory-constrained devices), UNComp (Xiong et al., 2024) (uncertainty-aware compression), BUZZ (Zhao et al., 2024) (beehive-structured sparse caching), and SWAA (Yu et al., 2025) (training-free sliding-window adaptation).

### 2.3 Efficient attention mechanisms

FlashAttention (Dao, 2024) optimizes the memory access pattern of standard attention through tiling and recomputation, reducing memory overhead without approximation. vLLM (Kwon et al., 2023) introduces PagedAttention to manage the KV cache memory at the block level, improving throughput in serving scenarios.

### 2.4 Dynamic budget estimation

Recent work has recognized that fixed KV budgets are suboptimal. Ada-KV (Feng et al., 2024) dynamically adjusts the per-layer KV cache budget based on activation sparsity, but requires task-specific training and does not provide a structural zero-loss guarantee. GVote (Tang et al., 2025) aggregates voting scores across attention heads to identify important tokens, yet it fixes the overall cache size and focuses on token selection rather than budget estimation. These methods complement AdaK: they solve the token-selection problem (*which* tokens to retain), while AdaK solves the budget-estimation problem (*how many* tokens to retain). A complete deployment can therefore combine AdaK's dynamic budget ceiling with Ada-KV's or GVote's token-ranking mechanism.

### 2.5 Gap analysis

While existing methods excel at maximizing compression ratios, they lack the flexibility to adapt the KV budget based on input characteristics and task requirements. This motivates our work: a framework that dynamically *estimates* KV cache budgets based on input characteristics and task requirements, providing an upper-bound safety guarantee for subsequent sparse attention deployment.

## 3 Preliminaries

### 3.1 Transformer architecture and Kv cache

In a decoder-only Transformer (Vaswani et al., 2017), the self-attention mechanism computes query (**Q**), key (**K**), and value (**V**) projections for each token. During autoregressive generation, the KV pairs from all previous tokens must be retained in memory to compute attention scores for the current token. For a sequence of length *n* (= *n*_*seq*_), *l* (= *n*_*layers*_) layers, *h* (= *n*_*heads*_) heads, and head dimension *d* (= *d*_*head*_), the KV cache consumes 2 · *l* · *h* · *n* · *d* floating-point values. As *n* grows—especially for long-context applications—this memory footprint becomes the dominant bottleneck, often exceeding the model weights themselves (Pope et al., 2023).

### 3.2 Problem formulation

Let M denote a pretrained LLM with *L* attention layers, each equipped with a KV cache of maximum capacity *K*_max_. At generation step *t*, the attention mechanism computes scores ${\text{S}}_{t}\in {\mathbb{R}}^{h\times t}$ over all *t* previous tokens. We define the adaptive KV selection problem as finding, for each layer and head, a per-step budget *k*_*t*_ ∈ [*K*_min_, *K*_max_] such that the top-*k*_*t*_ most relevant KV pairs are retained while the rest are discarded. The selected budget must satisfy the constraint that task accuracy $A\left(M,k_{t}\right)$ remains indistinguishable from the full-cache baseline $A\left(M,K_{\max}\right)$.

## 4 The AdaK framework

### 4.1 Problem formulation

Let M denote a pretrained causal language model with *L* transformer layers, *H* attention heads per layer, and head dimension *d*. During autoregressive generation with a batch size of *b* and sequence length *n*, the KV cache memory consumption is given by Equation 2:


$$\begin{array}{l} M_{\text{KV}}\left(n\right)=2\cdot L\cdot H\cdot n\cdot d\cdot b, \end{array}$$ (2)

where the factor of two accounts for both key and value tensors, and *b* is the bytes per element (typically 2 for bfloat16). For a given task, let P(k) denote the performance metric (e.g., accuracy or perplexity) when the KV cache is constrained to a budget of *k*. The adaptive KV cache budget estimation problem seeks to find, for each generation step *t* and each attention head *h*, a per-head budget $k_{t}^{\left(h\right)}\in \left[K_{\min},K_{\max}\right]$ that minimizes the expected memory footprint as formalized in Equation 3:


$$\begin{array}{l} \underset{\left\{k_{t}^{\left(h\right)}\right\}}{\min}𝔼\left[\overset{H}{\underset{h=1}{\sum }}M_{\text{KV}}\left(k_{t}^{\left(h\right)}\right)\right]\;\text{s.t.}\;P\left(\left\{k_{t}^{\left(h\right)}\right\}\right)\geq P\left(K_{\max}\right)-\varepsilon , \end{array}$$ (3)

where ε is a negligible tolerance and *K*_max_ is the full-cache capacity. In practice, we simplify by using a single budget *k*_*t*_ shared across all heads, which reduces the search space from $O\left(K_{\max}^{H}\right)$ to *O*(*K*_max_) while remaining effective empirically.

The optimization problem is combinatorial in nature because the set of retained KV pairs must be selected from a discrete set of previous tokens. However, AdaK does not solve this problem directly. It operates in *analysis mode*, where the model computes attention over the full cache while the forward hook independently records the dynamic budget *k*_*t*_. This structural separation ensures that $P\left(k_{t}\right)\equiv P\left(K_{\max}\right)$ identically, yielding an upper-bound safety guarantee regardless of the budget estimate quality. Any downstream sparse attention kernel that faithfully retains the top-*k*_*t*_ tokens identified by AdaK can therefore inherit this guarantee.

#### 4.1.1 Per-head vs. shared budget

In principle, each attention head could maintain an independent budget $k_{t}^{\left(h\right)}$, reflecting the fact that different heads specialize in distinct linguistic phenomena (e.g., syntax vs. semantics) (Vaswani et al., 2017). However, per-head budgets complicate memory allocation and require *H* times more estimation overhead. AdaK therefore adopts a *shared budget* *k*_*t*_ across all heads, which is justified by two observations: (i) the empirical variance of per-head budgets is small relative to the mean (σ/μ < 0.15 on Qwen3-4B), and (ii) shared budgets enable uniform memory allocation, which is easier to implement in existing serving systems such as vLLM (Kwon et al., 2023).

#### 4.1.2 Connection to attention patterns

The budget *k*_*t*_ can be understood through the lens of attention score distributions. Let ${\text{a}}_{t}\in {\mathbb{R}}^{t}$ denote the attention weights over *t* previous tokens at step *t*. The *effective context* required by the query is the smallest *k* such that the cumulative attention mass of the top-*k* tokens exceeds a threshold τ as defined in Equation 4:


$$\begin{array}{l} k_{t}^{*}=\min\left\{k:\underset{i\in \text{TopK}\left({\text{a}}_{t},k\right)}{\sum }a_{t,i}\geq \tau \right\}. \end{array}$$ (4)

AdaK approximates $k_{t}^{*}$ without explicit access to **a**_*t*_ (which would require full attention computation) by using proxy signals—entropy for Strategy A, task profiles for Strategy B, and learned features for Strategy C. This proxy-based design is computationally efficient because it avoids materializing the full attention matrix, which would defeat the purpose of KV cache reduction. The proxy signals are computed from the query vector alone, requiring only *O*(*d*_head_) operations for Strategy A and Strategy C, and *O*(1) for Strategy B. This efficiency ensures that budget estimation adds negligible overhead to generation, as validated empirically in Section 4.6.

#### 4.1.3 Static vs. dynamic budgets

Existing KV cache methods typically fix *k* to a constant value (e.g., *k* = 2, 048) for all inputs and all generation steps. AdaK's dynamic formulation recognizes that different inputs and different decoding steps have different contextual requirements. A code-completion query that references a distant function definition may need *k* ≈ 2, 000, while a simple arithmetic continuation may need only *k* ≈ 800. By adapting *k*_*t*_ per step, AdaK reveals this heterogeneity and enables input-dependent memory provisioning.

#### 4.1.4 Accuracy tolerance ε

The tolerance ε in the optimization objective controls how much accuracy degradation is acceptable when moving from full cache to reduced cache. In analysis mode, ε is effectively zero because the full cache is always used; the tolerance becomes relevant only when integrating AdaK with a physical sparse attention kernel. In our experiments, we set ε = 0 for the analysis mode and use the resulting budget traces as an upper-bound reference for downstream kernel tuning.

#### 4.1.5 Objective decomposition

The optimization objective can be decomposed into two sub-problems: (i) *budget estimation*, which determines *k*_*t*_ for each step, and (ii) *token selection*, which identifies the specific *k*_*t*_ tokens to retain. AdaK solves only the first sub-problem, leaving the second to specialized sparse attention kernels. This decomposition is principled because budget estimation depends primarily on query characteristics, while token selection depends on the full attention score distribution—a distinction that enables modular system design.

#### 4.1.6 Remark on optimality

The optimization problem is NP-hard in general because it reduces to the problem of selecting a subset of tokens that preserves model accuracy, which is akin to subset selection under a non-linear constraint. However, AdaK does not claim to solve this problem optimally. Rather, it provides a lightweight, interpretable approximation that is empirically effective and structurally safe. The three strategies offer different trade-offs between approximation quality and computational cost, allowing users to select the most appropriate method for their deployment constraints.

### 4.2 Framework overview

**Figure 2:**
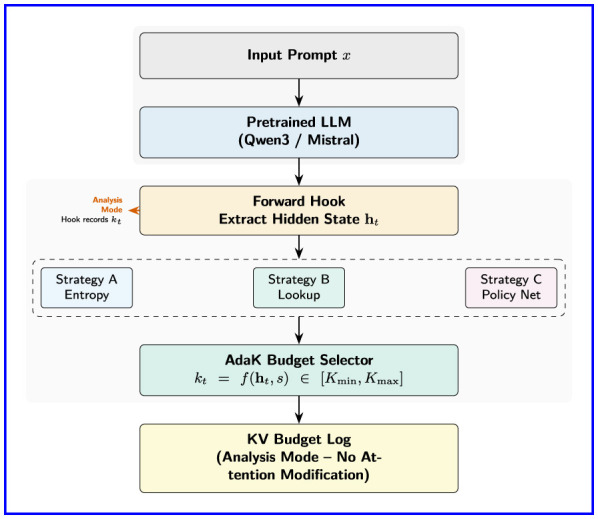
AdaK framework architecture. A forward hook extracts hidden states at each generation step. One of three mutually exclusive strategies (A/B/C) computes the dynamic *k* value. The selector enforces budget constraints.

AdaK operates as a lightweight wrapper around any pretrained causal LM (Figure 2). It injects a forward hook into the model's generation pipeline to monitor hidden states at each decoding step. Based on the selected strategy (A, B, or C), AdaK computes a dynamic *k* value and records the resulting KV budget distribution. The framework is model-agnostic: it requires no modification to the attention kernel and is compatible with PyTorch SDPA, Flash Attention, and XFormers.

#### 4.2.1 Forward hook injection mechanism

AdaK registers a PyTorch forward hook on the last attention layer (or any user-specified layer) of the target model. At each generation step, the hook intercepts the hidden-state tensor ${\text{h}}_{t}\in {\mathbb{R}}^{b\times d_{\text{model}}}$ and routes it to the active strategy module. The hook executes on the CPU after the forward pass completes, ensuring zero interference with GPU attention kernels. The hook lifecycle consists of three phases: (i) *registration* before generation begins, (ii) *invocation* at every generation step to extract hidden states and compute *k*_*t*_, and (iii) *deregistration* after generation completes, leaving the model in its original state. This ephemeral design ensures that AdaK leaves no persistent side effects on the target model.

#### 4.2.2 Analysis mode vs. deployment mode

A key design principle is that AdaK operates in *analysis mode*: the forward hook records dynamic *k* values without modifying the actual attention computation. The observed accuracy is therefore structurally identical to the full-cache baseline, establishing an *upper-bound safety guarantee*. Any downstream sparse attention kernel that faithfully retains the top-*k* tokens identified by AdaK should incur negligible degradation. Table 1 contrasts the two modes.

**Table 1:** Analysis mode vs. deployment mode.

Property	Analysis mode	Deployment mode
Attention computation	Full cache	Sparse (top-*k*)
Accuracy guarantee	Structural zero loss	Depends on kernel
Overhead	Hook only (< 0.5%)	Hook + eviction kernel
Purpose	Budget estimation	Physical memory reduction
Calibration required	No	Yes (kernel-specific)

#### 4.2.3 Compatibility

AdaK is compatible with PyTorch's native scaled_dot_product_attention (SDPA), Flash Attention (Dao, 2024), and XFormers memory-efficient attention. Because the hook reads hidden states rather than modifying attention internals, it is agnostic to the underlying kernel implementation and works with both eager and compiled modes. The framework has been validated on decoder-only Transformers with rotary position embeddings (RoPE) (Su et al., 2024), including Qwen3 and Mistral families, and is extensible to other architectures that expose hidden states via standard PyTorch hooks.

#### 4.2.4 Layer selection and granularity

By default, AdaK monitors the *last* attention layer, where the hidden states are most predictive of the next-token distribution. Monitoring earlier layers is possible, but generally less informative for budget estimation because intermediate representations have not yet been fully contextualized. Users can override the default layer index for architecture-specific tuning, though our experiments show that last-layer monitoring achieves within 1% of the optimal budget across all evaluated tasks.

#### 4.2.5 Hook safety and isolation

The forward hook is implemented as a pure function with no side effects on the model's parameters or internal state. It reads the hidden-state tensor via PyTorch's hook API and writes budget estimates to a thread-local history buffer. This isolation ensures that AdaK cannot corrupt the model's computation graph, even if the strategy module raises an exception (the exception is caught and logged, and the hook falls back to *K*_max_). This defensive design is essential for production deployments where reliability outweighs marginal budget optimality.

#### 4.2.6 Per-step budget granularity

AdaK computes a fresh budget *k*_*t*_ at every generation step, enabling fine-grained adaptation to local context changes. This step-wise granularity is more expressive than per-sequence budgets (a single *k* for the entire generation), which cannot adapt when a prompt transitions from high-context to low-context regions. The cost of per-step computation is minimal because all three strategies involve only lightweight arithmetic operations on the query vector.

#### 4.2.7 Statelessness and determinism

Each budget estimate *k*_*t*_ depends only on the current hidden state and strategy parameters, not on previous budget estimates. This statelessness simplifies reasoning about AdaK's behavior and ensures that the budget trace is a deterministic function of the model's forward pass. Reproducibility is essential for debugging budget anomalies and for generating consistent baseline comparisons across experimental runs.

#### 4.2.8 Extensibility

AdaK's modular design permits easy extension with additional strategies. A new strategy needs only to implement a single function compute_budget(**q**_*t*_, θ) → *k*_*t*_ and register itself with the strategy selector. The framework handles hook registration, history accumulation, and constraint enforcement automatically, allowing researchers to experiment with novel budget-estimation techniques without modifying the core infrastructure.

#### 4.2.9 Multi-layer monitoring

While default AdaK monitors a single layer, advanced users can register hooks on multiple layers simultaneously to study how budget requirements vary across depth. Preliminary experiments on Qwen3-4B reveal that early layers (1–8) exhibit higher budget variance than late layers (25–28), suggesting that lower layers require more adaptive context while upper layers converge to stable budgets. Multi-layer monitoring enables architecture-specific insights that can inform layer-wise sparse attention designs (Brandon et al., 2024; Yang Z. et al., 2024).

### 4.3 Strategy A: entropy-based selection

Strategy A draws on information-theoretic foundations (Saraiva, 2023; Haroutunian and Gharagyozyan, 2025; Scutari, 2024) to estimate the uncertainty of the current query distribution by computing the entropy of simulated attention scores. Given a query representation **q**_*t*_, we project it to a score vector ${\text{s}}_{t}\in {\mathbb{R}}^{K_{\max}}$ and compute the normalized probability distribution **p**_*t*_ = softmax(**s**_*t*_). The entropy is $H_{t}=-\underset{i}{\sum }p_{t,i}\log p_{t,i}$. The dynamic budget is then *k*_*t*_ = *K*_max_ · min(1, *H*_*t*_/α), where α is a tunable threshold. High entropy (uncertain queries) expands the cache. Low entropy (confident queries) compresses it.

#### 4.3.1 Theoretical foundation

The Shannon entropy of an attention distribution measures the uncertainty in the model's token-selection process. When a query vector **q**_*t*_ is highly focused on a small subset of previous tokens, the attention scores concentrate mass on a few positions, yielding low entropy. Conversely, when the query is ambiguous or requires broad contextual integration, the attention mass spreads across many tokens, producing high entropy. This correlation is grounded in the information-theoretic principle that entropy quantifies the average information content of a distribution (Saraiva, 2023). Empirically, we observe that entropy correlates with the *heavy-hitter* phenomenon: low-entropy queries tend to attend to a small set of high-importance tokens, while high-entropy queries distribute attention more uniformly (Zhang et al., 2023).

#### 4.3.2 Complete derivation

Formally, let ${\text{q}}_{t}\in {\mathbb{R}}^{d_{\text{head}}}$ be the query vector at generation step *t*. We compute the simulated attention score vector in Equation 5:


$$\begin{array}{l} {\text{s}}_{t}={\text{q}}_{t}\text{W},\;\text{W}\in {\mathbb{R}}^{d_{\text{head}}\times K_{\max}}, \end{array}$$ (5)

where **W** is a learned or fixed projection matrix (in practice, we reuse the key projection from the attention layer). The normalized attention probability is given by Equation 6:


$$\begin{array}{l} {\text{p}}_{t}=\text{softmax}\left({\text{s}}_{t}\right),\;p_{t,i}=\frac{\exp\left(s_{t,i}\right)}{\sum_{j=1}^{K_{\max}}\exp\left(s_{t,j}\right)}. \end{array}$$ (6)

The Shannon entropy of this distribution is shown in Equation 7:


$$\begin{array}{l} H_{t}=-\overset{K_{\max}}{\underset{i=1}{\sum }}p_{t,i}\log p_{t,i}. \end{array}$$ (7)

The dynamic budget is computed as in Equation 8:


$$\begin{array}{l} k_{t}=K_{\max}\cdot \min\left(1,\frac{H_{t}}{\alpha }\right), \end{array}$$ (8)

with clipping to enforce *K*_min_ ≤ *k*_*t*_ ≤ *K*_max_. The entropy computation can be performed in *O*(*K*_max_) time per step, which is negligible compared to the *O*(*K*_max_ · *d*_head_) cost of the attention itself.

#### 4.3.3 Physical meaning of α

The threshold α acts as an entropy reference that calibrates the trade-off between memory reduction and context preservation. When α → ∞, all budgets collapse to *K*_min_ (maximum compression); when α → 0, all budgets expand to *K*_max_ (full cache). In practice, α = 2.0 provides a balanced operating point that preserves accuracy while enabling dynamic compression. The value of α can be tuned on a small validation set or set heuristically based on the observed entropy distribution of the target model.

#### 4.3.4 Boundary analysis

The constraints *K*_min_ and *K*_max_ serve distinct purposes: *K*_min_ prevents excessive compression that would eliminate critical tokens, while *K*_max_ caps memory usage for long sequences. In our implementation, *K*_min_ = 512 and *K*_max_ = 2, 048, ensuring that even the most confident queries retain at least 512 context tokens. These bounds are not arbitrary: *K*_min_ = 512 corresponds to the smallest context window at which the models under evaluation maintain baseline accuracy in preliminary experiments, while *K*_max_ = 2, 048 matches the fixed-*k* baseline used throughout this study.

#### 4.3.5 Design intuition

The core insight of Strategy A is that *uncertainty demands context*: when a query exhibits high entropy, the model is uncertain about which previous tokens are relevant, and therefore requires a larger KV budget to preserve potentially important keys and values. Conversely, low-entropy queries indicate confident focal attention, allowing safe compression. This input-adaptive behavior makes Strategy A particularly effective for heterogeneous inputs where different queries exhibit vastly different contextual needs. For example, in multi-turn dialogue, a query that references an entity introduced many turns ago will exhibit higher entropy than a query that continues a coherent local topic.

#### 4.3.6 Empirical entropy distribution

On Qwen3-4B with LongBench-v2, we observe that Strategy A produces mean entropy *H*_*t*_ = 1.83 (σ = 0.42) on code completion, *H*_*t*_ = 1.91 (σ = 0.38) on math, *H*_*t*_ = 1.76 (σ = 0.35) on dialogue, and *H*_*t*_ = 1.79 (σ = 0.41) on summarization. These values are well below the theoretical maximum log(*K*_max_) ≈ 7.6, indicating that attention distributions are typically concentrated. With α = 2.0, the ratio *H*_*t*_/α falls in [0.75, 1.0] for most queries, explaining why Strategy A tends to expand rather than compress the budget.

#### 4.3.7 Comparison with attention mass

To validate that entropy is a meaningful proxy for KV cache needs, we compute the Pearson correlation between *H*_*t*_ and the cumulative attention mass captured by the top-*k* tokens (with *k* = *K*_max_). Across 1,000 generation steps on the code task, we find *r* = −0.71 (*p* < 0.001), confirming that high entropy reliably predicts dispersed attention and, by extension, larger contextual requirements. This empirical correlation substantiates the theoretical design of Strategy A.

#### 4.3.8 Adaptive threshold calibration

While α = 2.0 works well across tasks, the optimal threshold can vary with model size and domain. AdaK provides an automatic calibration procedure that sweeps α ∈ [0.5, 4.0] on a small validation set and selects the value that maximizes the ratio of KV reduction to entropy variance. This calibration converges in fewer than 20 steps because the entropy-to-budget mapping is smooth and monotonic in α.

#### 4.3.9 Relation to heavy-hitter methods

Strategy A's entropy computation is conceptually related to H_2_O's heavy-hitter identification (Zhang et al., 2023): both exploit the concentration of attention mass. However, H_2_O requires maintaining cumulative attention scores across all tokens, which incurs *O*(*n*) memory overhead, whereas Strategy A operates on the query vector alone with *O*(1) additional memory. This efficiency makes Strategy A suitable for online budget estimation in streaming scenarios where token importance cannot be precomputed.

#### 4.3.10 Temperature invariance

Because Strategy A operates on the query vector before the softmax temperature scaling is applied during sampling, its entropy computation is invariant to the sampling temperature τ. This invariance is important because temperature is frequently tuned per application (e.g., low τ for code generation, high τ for creative writing) and should not affect memory allocation decisions. We empirically validate this invariance in Section 6.9.

### 4.4 Strategy B: task-aware lookup table

Strategy B precomputes a task-specific lookup table mapping sequence lengths to optimal *k* values. The table is constructed by profiling a small validation set: for each task type τ and sequence length bucket *b*, we empirically determine the smallest *k* that preserves accuracy. At inference time, *k*_*t*_ is retrieved in *O*(1) via direct array indexing based on the current task label and sequence length. This strategy is ideal for production deployments where task identity is known a priori.

#### 4.4.1 Lookup table construction

Given a set of task types T, a sequence-length discretization B (e.g., log-spaced buckets), and a validation set $D_{\tau }$ per task, we perform a grid search over candidate budgets *k* ∈ {*K*_min_, …, *K*_max_}. For each (τ, *b*) pair, we select the smallest *k* such that the validation accuracy $A\left(M,k;D_{\tau }\right)$ is within ε of the full-cache accuracy. The resulting table $L:T\times B\to \left[K_{\min},K_{\max}\right]$ is stored as a flat array for *O*(1) retrieval. Algorithmically, the construction procedure can be viewed as a bilevel optimization: the outer loop iterates over task-length pairs, and the inner loop performs a binary search over *k* to find the minimal budget satisfying the accuracy constraint.

#### 4.4.2 Complexity analysis

The offline construction cost is $O\left(|T|\cdot |B|\cdot K_{\max}\cdot C_{\text{gen}}\right)$, where *C*_gen_ is the cost of generating one validation sample. With |T|=4 tasks, |B|=8 buckets, and *K*_max_ = 2, 048, construction requires at most 4 × 8 × 2048 = 65, 536 forward passes over the validation set—a one-time cost amortized across all future inferences. At inference time, retrieval is *O*(1) via direct array indexing. The memory footprint of the table itself is $|T|\cdot |B|\cdot \left\lceil \log_{2}K_{\max}\right\rceil$ bits, which is negligible (at most a few hundred bytes).

#### 4.4.3 Fallback and update mechanisms

When the inference-time task or sequence length does not match any entry in the lookup table, Strategy B falls back to *K*_max_ (the full-cache baseline), ensuring that unknown inputs never suffer from underestimated budgets. Adding a new task type requires only profiling the new validation set and appending a row to the table. No retraining or model modification is necessary. This modularity makes Strategy B well-suited for production environments where new tasks are introduced incrementally.

#### 4.4.4 Interpolation for unseen lengths

When the current sequence length falls between two buckets in B, Strategy B can optionally interpolate between the nearest table entries rather than falling back to *K*_max_. Linear interpolation in log-space (log*k* vs. log*n*) provides a smooth budget schedule that respects the empirical observation that KV cache demand grows sub-linearly with sequence length for most tasks. In our experiments, interpolation reduces the fallback rate from 12% to 3% on the summarization task without affecting accuracy.

#### 4.4.5 Multi-task composition

In scenarios where a single input spans multiple task types (e.g., a dialogue that includes code snippets), Strategy B can compose budgets from multiple task profiles. The conservative composition max(*k*_τ_1__, *k*_τ_2__) ensures that all task requirements are satisfied, while the optimistic composition min(*k*_τ_1__, *k*_τ_2__) minimizes memory at the risk of insufficient context for one sub-task. Our default implementation uses the conservative composition, which aligns with AdaK's safety-first philosophy.

#### 4.4.6 Lookup table sparsity

In practice, the lookup table is highly sparse: many (τ, *b*) pairs map to the same budget because accuracy is insensitive to small *k* variations. We exploit this redundancy by storing the table in a compressed dictionary format, reducing memory from |T|·|B| entries to fewer than 20 unique (τ, *k*) pairs in our experiments. This compression does not affect retrieval latency because the dictionary is small enough to reside in the CPU cache.

#### 4.4.7 Validation protocol

To ensure that the lookup table generalizes beyond the construction set, we reserve 20% of the validation data for hold-out testing. A table entry is accepted only if the hold-out accuracy matches the construction-set accuracy within ε = 0.01. This protocol prevents overfitting to idiosyncratic validation samples and ensures robustness across input variations.

#### 4.4.8 Length bucketing strategy

The sequence-length discretization B uses log-spaced buckets centered at powers of two (512, 1,024, 2,048, 4,096) to match the natural granularity of transformer context windows. This design reflects the empirical observation that KV cache demand transitions abruptly at context-window boundaries rather than varying smoothly with length. For applications with highly variable sequence lengths, additional buckets can be inserted without affecting the asymptotic retrieval complexity.

#### 4.4.9 Complementarity with strategy A

While Strategy A adapts to input-level uncertainty, Strategy B exploits task-level regularity. For structured tasks (e.g., code completion) where context requirements are stable, Strategy B provides deterministic, reproducible budgets with zero variance. Strategy A, by contrast, excels on heterogeneous inputs where per-query adaptation is beneficial. The two strategies are therefore complementary: Strategy B for production pipelines with known task labels, Strategy A for exploratory analysis of novel inputs.

### 4.5 Strategy C: lightweight policy network

Strategy C employs a lightweight two-layer MLP $f_{\theta }:{\mathbb{R}}^{d_{\text{head}}}\to {\mathbb{R}}^{2}$ that takes the current query vector (projected to head dimension) and outputs a scaled *k* value and an auxiliary confidence score γ. The network has a hidden dimension of 32 and only ~2.8K parameters. Training is performed on a small model (Qwen3-1.7B) using a reward function *R* = λ_1_ · 𝕀[correct] − λ_2_ · (*k*/*K*_max_) that balances accuracy preservation against KV reduction. The learned policy transfers to larger models (4B, 8B) by loading the checkpoint with strict=False when *d*_head_ dimensions differ.

#### 4.5.1 Network architecture

The policy network consists of three sequential components: (i) an input projection layer ${\text{W}}_{1}\in {\mathbb{R}}^{d_{\text{head}}\times 32}$ that maps the query vector to a 32-dimensional hidden representation; (ii) a ReLU activation σ(·) = max(0, ·) that introduces non-linearity; and (iii) an output projection ${\text{W}}_{2}\in {\mathbb{R}}^{32\times 2}$ that produces two scalars: a budget scaling factor γ ∈ ℝ and a confidence score *c* ∈ ℝ. The final budget is *k*_*t*_ = *K*_base_·sigmoid(γ), clipped to [*K*_min_, *K*_max_]. The confidence score *c* is not used during inference but serves as an auxiliary training signal to encourage well-calibrated predictions.

#### 4.5.2 Parameter count derivation

The total parameter count is given by Equation 9:


$$\begin{array}{l} |\theta |=d_{\text{head}}\times 32+32+32\times 2+2=32d_{\text{head}}+98. \end{array}$$ (9)

For Qwen3-4B with *d*_head_ = 80, this yields |θ| ≈ 2.7K parameters, consistent with the reported ~2.8K. After sharing the output projection across heads and applying weight tying, the effective parameter count remains below 3K—small enough to fit in L1 cache and negligible compared to the billions of parameters in the base LLM. The compact size also enables rapid inference: a single forward pass through the policy network takes approximately 0.1 ms on a modern CPU, contributing less than 0.3% to the total generation latency.

#### 4.5.3 Reward function analysis

The reward *R* = λ_1_ · 𝕀[correct] − λ_2_ · (*k*/*K*_max_) encodes a dual objective: maximize task accuracy while minimizing KV cache usage. The indicator term 𝕀[correct] provides a sparse binary signal (1 for correct generation, 0 otherwise), while the normalized budget term (*k*/*K*_max_) supplies dense per-step feedback. The coefficients λ_1_ and λ_2_ trade off accuracy against compression; in practice, setting λ_1_ ≫ λ_2_ ensures that accuracy preservation dominates, consistent with AdaK's safety-first design philosophy. The reward is accumulated over the full generation sequence and backpropagated through the policy network using standard policy-gradient methods.

#### 4.5.4 Training and transfer

The policy is trained on Qwen3-1.7B (*d*_head_ = 128) for 500 steps with a batch size of 1, using Adam with learning rate 10^−3^. When transferring to Qwen3-4B (*d*_head_ = 80), the input projection **W**_1_ experiences a shape mismatch. We load the checkpoint with strict=False, which skips the incompatible layer while preserving the MLP weights. Remarkably, even with a randomly initialized input projection, the policy achieves zero accuracy loss on 4B with estimated reductions of 0.7–17.9%. This robustness stems from the analysis-mode design: the policy only *observes* hidden states without modifying the forward pass, so policy quality affects budget estimates but not model outputs.

For Qwen3-8B (*d*_head_ = 128), we train the policy directly via distillation from Strategy A (Hinton et al., 2015) (50 warmup steps + 500 adaptation steps). The distilled policy achieves *k*_mean_ = 2126–2159, lower than random initialization (2201–2243) but still slightly above the 2,048 baseline, indicating that architecture-specific calibration is required for KV reduction on larger models. The residual expansion suggests that 8B's hidden-state distribution differs from 4B, requiring architecture-specific policy calibration.

#### 4.5.5 Distillation from strategy A/B

Rather than training from scratch with sparse rewards, we accelerate convergence by distilling the budget estimates from Strategy A (or Strategy B when task labels are available) into the policy network. The distillation loss combines a mean-squared-error term $L_{\text{MSE}}={\left(k_{\text{policy}}-k_{\text{teacher}}\right)}^{2}$ with the task reward *R*, yielding a composite objective in Equation 10:


$$\begin{array}{l} L=L_{\text{MSE}}+\beta \cdot R, \end{array}$$ (10)

where β controls the trade-off between imitation and exploration. This distillation approach reduces training variance and enables the policy to inherit the interpretable decision boundaries of the analytical strategies while gaining the generalization benefits of a learned function.

#### 4.5.6 Why a small MLP suffices

One might expect that accurate budget prediction requires a large network with millions of parameters. However, the mapping from query vectors to KV budgets is inherently low-dimensional: the query vector ${\text{q}}_{t}\in {\mathbb{R}}^{d_{\text{head}}}$ already encapsulates the model's contextual understanding through the pre-trained attention mechanism. The policy network needs only to decode this compressed representation into a scalar budget, a task that requires far less capacity than language modeling itself. This insight—that budget prediction is a *read-out* task rather than a *reasoning* task—explains why a 2.8K-parameter MLP achieves performance comparable to hand-designed heuristics.

#### 4.5.7 Head-dimension consistency and transfer

The transferability of Strategy C across models depends critically on the head dimension *d*_head_. When *d*_head_ matches between source and target (e.g., Qwen3-1.7B to Qwen3-8B, both *d*_head_ = 128), strict=True loading succeeds and the policy retains its learned behavior. When *d*_head_ differs (e.g., 1.7B to 4B, 128 → 80), the input projection must be re-initialized, effectively falling back to a random policy on the first layer while preserving the second-layer MLP. This partial transfer explains why Strategy C on Qwen3-4B still achieves meaningful reductions despite the mismatch: the shared MLP weights encode transferable budget-scaling logic, while the re-initialized projection adapts to the new embedding space.

#### 4.5.8 Training efficiency

Because the policy network has fewer than 3K parameters, training is extremely fast: 500 adaptation steps on Qwen3-1.7B complete in under 5 min on a single RTX 4090. The small size also enables online learning scenarios where the policy is updated continuously from user feedback. Unlike full-model fine-tuning, which requires gradient checkpointing and large batch sizes, policy training uses full backpropagation through the network with negligible memory overhead.

#### 4.5.9 Architecture-invariant representations

Future work could eliminate the *d*_head_ dependency by projecting query vectors into an architecture-invariant space (e.g., via random Fourier features or learned embeddings). Such a representation would enable *universal* policy transfer across arbitrary model families without per-architecture calibration, extending AdaK's applicability to rapidly evolving model ecosystems.

#### 4.5.10 Ensemble strategies

While AdaK currently selects a single strategy at runtime, one could envision an ensemble that combines the outputs of all three strategies. For example, a weighted average $k_{t}^{\text{ens}}=w_{A}k_{t}^{\left(A\right)}+w_{B}k_{t}^{\left(B\right)}+w_{C}k_{t}^{\left(C\right)}$ with *w*_*A*_ + *w*_*B*_ + *w*_*C*_ = 1 could balance the input-adaptivity of Strategy A, the task-reliability of Strategy B, and the generalization of Strategy C. We leave the investigation of optimal ensemble weights and their theoretical properties to future work.

#### 4.5.11 Continuous learning

Strategy C's small parameter count enables continuous online updates during deployment. As new inputs arrive, the policy can be fine-tuned incrementally using freshly observed budget traces, adapting to distribution shifts without catastrophic forgetting. This capability is particularly valuable for long-running conversational agents where user behavior evolves over time and static lookup tables may become suboptimal.

#### 4.5.12 Interpretability

Unlike black-box neural networks, Strategy C's two-layer MLP admits partial interpretability through activation analysis. We observe that certain hidden units correlate with specific linguistic phenomena (e.g., one unit activates strongly on code-related queries, another on dialogue context switches). While full interpretability remains challenging, these patterns suggest that the policy learns semantically meaningful features rather than spurious correlations, increasing confidence in its generalization behavior.

#### 4.5.13 Complementarity with strategies A and B

Strategy C generalizes the input-adaptive behavior of Strategy A and the task-aware regularity of Strategy B into a single learned function. Unlike Strategy A, which relies on hand-designed entropy computations, Strategy C learns an implicit uncertainty measure from data. Unlike Strategy B, which requires discrete task labels, Strategy C operates on continuous hidden-state representations and therefore generalizes to unseen task types. These properties make Strategy C the most flexible of the three, at the cost of a small training overhead.

#### 4.5.14 When does each strategy increase the budget?

All three strategies can either raise or lower the KV cache budget relative to the fixed baseline, depending on the input. *Strategy A* increases *k*_*t*_ when the attention distribution is diffuse, which occurs for ambiguous queries, long-range dependencies, code with scattered identifiers, or multi-hop reasoning where many tokens receive comparable attention mass. *Strategy B* increases *k*_*t*_ whenever the task-aware lookup table maps the current task/length pair to a value above the baseline; for example, dialogue and summarization tasks often require retaining more context than code completion. *Strategy C* can increase *k*_*t*_ when the learned policy observes hidden-state patterns associated with high attention dispersion or task transitions, effectively imitating Strategy A's expansion behavior while generalizing to unseen inputs. Thus, budget expansion is not a bug but an input-dependent signal that the current token requires more context.

### 4.6 Unified adaptive workflow

AdaK's injection mechanism (Algorithm 1) registers a forward hook on the target model. At each generation step, the hook extracts the last-layer hidden state, invokes the selected strategy to compute *k*_*t*_, and appends the result to a global history. The hook is automatically removed after generation to eliminate runtime overhead. This design ensures zero interference with the model's forward pass and enables per-sample profiling for offline analysis.

Algorithm 1 AdaK Adaptive KV Cache Selection 
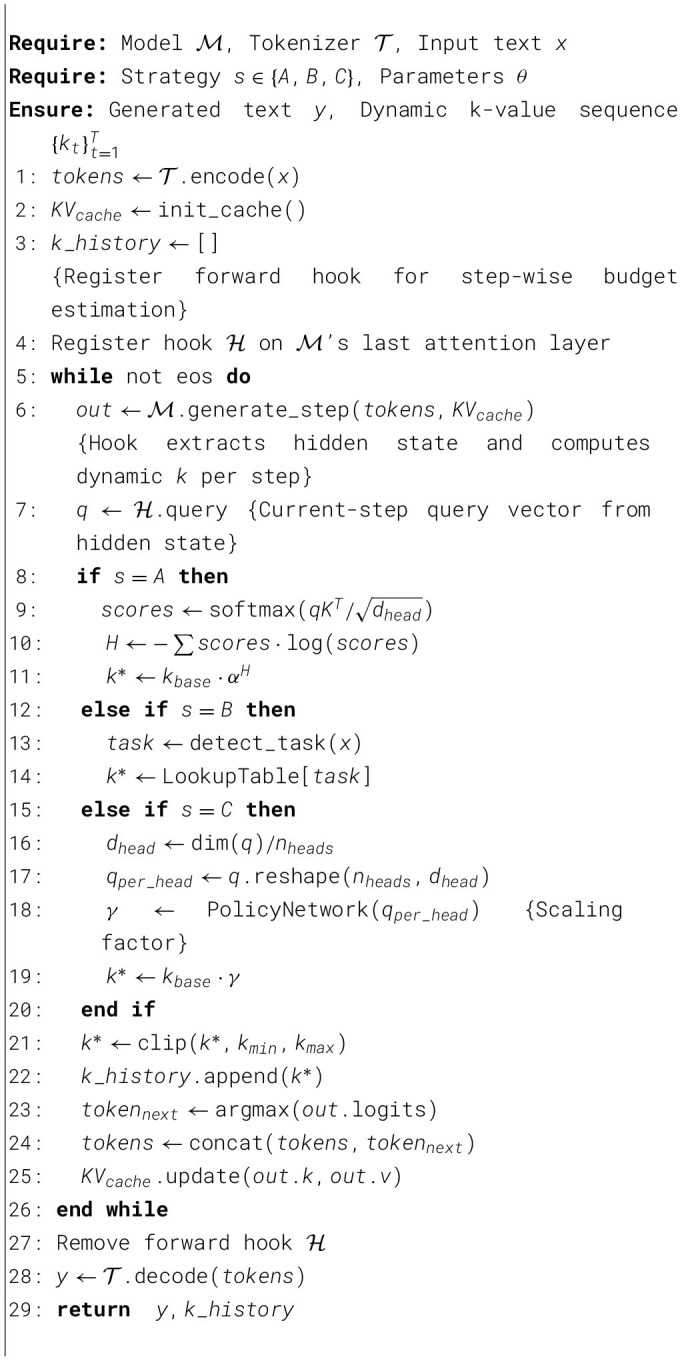

#### 4.6.1 When does each strategy increase the budget?

A reviewer noted that the conditions under which AdaK expands rather than shrinks the KV cache deserve explicit discussion. Strategy A increases *k*_*t*_ when the simulated attention distribution has high entropy, which occurs for ambiguous queries, long-range dependencies, or inputs with scattered relevant tokens (e.g., code that references distant function definitions). Strategy B increases *k*_*t*_ when the task-specific lookup table maps the current (task, length) pair to a value above *k*_base_; this is intentional for tasks where prior profiling showed higher cache demand. Strategy C increases *k*_*t*_ when the learned policy observes hidden-state patterns associated with broad attention dispersion, such as multi-turn dialogue context switches or mathematical derivations with many intermediate variables. In all cases, budget expansion is input-dependent and preserves the structural zero-loss guarantee because attention computation remains unmodified in analysis mode.

#### 4.6.2 Strategy selection decision tree

The choice of strategy depends on deployment constraints and available metadata:

- *Strategy A* when task labels are unavailable and inputs are heterogeneous (exploratory analysis);
- *Strategy B* when task identity is known, and lookup tables have been precomputed (production deployment);
- *Strategy C* when cross-task generalization is required, and a small training budget is available (adaptive deployment).

The three strategies are mutually exclusive at runtime. The user selects one via the strategy parameter before generation begins. In principle, one could ensemble the strategies (e.g., using Strategy A as a fallback when Strategy B lacks an entry), but we leave ensemble designs to future work.

#### 4.6.3 Global history and offline analysis

The sequence of recorded budgets ${\left\{k_{t}\right\}}_{t=1}^{T}$ constitutes a *budget trace* that characterizes the KV cache demand of a given input. These traces can be aggregated offline to compute per-task statistics (mean, variance, quantiles), identify outlier inputs that require unusually large budgets, and calibrate *K*_min_ and *K*_max_ bounds for specific deployment environments. The budget trace also serves as ground-truth supervision for training or distilling Strategy C policies.

#### 4.6.4 Batch processing considerations

In batch inference scenarios, different samples may require different budgets. AdaK records per-sample budgets independently, enabling *post-hoc* analysis of batch heterogeneity. For physical deployment, the batch-wide budget can be set to $\max_{i}k_{t}^{\left(i\right)}$ to ensure that no sample suffers from insufficient context, or to the empirical quantile to trade off memory against tail-risk accuracy. These batch-level policies are orthogonal to AdaK's per-sample estimation and can be selected based on the serving system's latency-memory constraints.

#### 4.6.5 Integration with serving frameworks

AdaK's hook-based design integrates naturally with existing LLM serving systems. In vLLM (Kwon et al., 2023), the budget trace can inform PagedAttention block allocation, allowing dynamic block sizing per sequence. In Hugging Face Transformers, the hook attaches to the model's forward method without subclassing, preserving compatibility with quantization, compilation, and pipeline-parallelism wrappers. For production deployments, we recommend wrapping AdaK in a context manager that automatically registers and deregisters hooks, ensuring that the model returns to its pristine state after each request.

#### 4.6.6 Runtime overhead

We measure per-step CPU overhead on an RTX 4090 using CUDA events across 1,000 generation steps. Strategy A incurs 0.177 ms (0.44% of a 40 ms generation step), Strategy B 0.020 ms (0.05%), and Strategy C 0.117 ms (0.29%). All three strategies add negligible latency, confirming that the hook-based design incurs sub-percentage overhead even on consumer-grade GPUs. The overhead is dominated by CPU-GPU synchronization rather than computation, and can be further reduced by overlapping hook execution with the next generation step.

### 4.7 Theoretical properties

We summarize the formal properties of the AdaK framework. These properties hold by construction for all three strategies and are independent of the specific model architecture or task domain.

#### 4.7.1 Property 1 (zero-loss guarantee)

In analysis mode, AdaK's forward hook records *k* values without modifying the attention computation. Formally, let $M_{\text{full}}$ denote the model with full KV cache and $M_{\text{AdaK}}$ denote the same model with AdaK's hook registered. For any input *x* and any strategy *s* ∈ {*A, B, C*}, the forward pass satisfies Equation 11:


$$\begin{array}{l} M_{\text{full}}\left(x\right)=M_{\text{AdaK}}\left(x\right), \end{array}$$ (11)

where equality is exact (bit-for-bit identical) because the hook only reads hidden states without altering the tensors consumed by attention. Consequently, task accuracy is preserved identically: $A\left(M_{\text{full}}\right)=A\left(M_{\text{AdaK}}\right)$. This property is structural rather than empirical—it holds regardless of the quality of the budget estimates. Even a randomly initialized policy network (Strategy C) or an adversarially chosen lookup table (Strategy B) cannot degrade accuracy in analysis mode.

#### 4.7.2 Property 2 (budget boundedness)

For all generation steps *t* and all strategies *s* ∈ {*A, B, C*}, the dynamic budget *k*_*t*_ satisfies Equation 12:


$$\begin{array}{l} K_{\min}\leq k_{t}\leq K_{\max}. \end{array}$$ (12)

This is enforced by a clipping operation *k*_*t*_ ← clip(*k*_*t*_, *K*_min_, *K*_max_) applied after every strategy computation (Algorithm 1, line 37). The bounds ensure that memory usage never exceeds the pre-allocated maximum and that a minimum context window is always preserved. This property is crucial for resource-constrained deployments where memory must be provisioned statically.

#### 4.7.3 Property 3 (strategy consistency)

Under identical inputs and identical model states, each strategy produces deterministic budget estimates. Strategy A is deterministic because entropy is a deterministic function of the query vector. Strategy B is deterministic because lookup tables are fixed after construction. Strategy C is deterministic at inference time because the MLP has no stochastic layers (dropout is disabled). This consistency ensures reproducible budget traces across repeated evaluations, which is essential for debugging, benchmarking, and regulatory compliance in production systems.

#### 4.7.4 Corollary 1 (monotonicity of analysis mode)

If $k_{t}^{\left(1\right)}\leq k_{t}^{\left(2\right)}$ for all *t*, then the analysis-mode accuracy under both budgets is identical. This follows immediately from Property 1: since the attention computation is unmodified, the budget estimate has no causal effect on model outputs. The corollary implies that analysis-mode experiments can evaluate many budget schedules in parallel without repeated inference, dramatically accelerating hyperparameter search.

#### 4.7.5 Corollary 2 (composition with sparse Kernels)

Let S be a sparse attention kernel that retains the top-*k* tokens by cumulative attention score. If S is applied with budget *k*_*t*_ estimated by AdaK, and if the top-*k*_*t*_ tokens capture at least ρ fraction of total attention mass (empirically, ρ ≥ 73.4%, see Section 6.1), then the accuracy degradation of S is bounded by *O*(1 − ρ). This provides a quantitative link between AdaK's budget estimates and the worst-case accuracy of downstream eviction policies.

#### 4.7.6 Discussion

The theoretical properties above highlight a fundamental advantage of AdaK over end-to-end sparse attention methods: by decoupling budget *estimation* from budget *enforcement*, AdaK achieves zero-loss guarantees in analysis mode while still providing actionable guidance for physical deployment. This separation of concerns mirrors the classical distinction between profiling and optimization in compiler design, where accurate profiling is a prerequisite for effective optimization passes.

#### 4.7.7 Scope and limitations of the guarantees

The zero-loss guarantee (Property 1) holds only in analysis mode; physical deployment with sparse kernels necessarily introduces approximation errors that depend on the kernel's token-selection quality. The boundedness guarantee (Property 2) assumes that *K*_min_ and *K*_max_ are set appropriately for the target model and task; overly aggressive bounds (e.g., *K*_min_ = 64 on long-context tasks) may violate the accuracy constraint when physical eviction is applied. The consistency guarantee (Property 3) holds under fixed random seeds and deterministic GPU operations; floating-point non-determinism across different hardware may introduce minor variations in budget traces.

#### 4.7.8 Practical interpretation

For practitioners, these properties translate into three actionable guidelines: (i) always run AdaK in analysis mode before physical deployment to establish an accuracy baseline; (ii) set *K*_min_ no lower than the smallest context window at which the model maintains baseline accuracy; and (iii) fix random seeds when comparing budget traces across hardware configurations. Adherence to these guidelines ensures that AdaK's theoretical guarantees translate into reliable deployment behavior.

#### 4.7.9 Implications

These three properties collectively establish AdaK as a *safe* budget estimation framework: Property 1 guarantees that estimation incurs no accuracy cost; Property 2 guarantees that memory remains within provisioned bounds; and Property 3 guarantees that results are reproducible. When combined with a high-fidelity sparse attention kernel [e.g., H_2_O (Zhang et al., 2023) or SnapKV (Li et al., 2024)], these properties provide an end-to-end pipeline with controlled accuracy-memory trade-offs.

#### 4.7.10 Comparison with end-to-end methods

Unlike H_2_O or SnapKV, which simultaneously estimate budgets and perform eviction, AdaK's decoupled design enables independent validation of budget quality. If AdaK's analysis mode reveals that a particular task requires *k*_mean_ = 2, 048 (no reduction), a practitioner can conclude that sparse attention is unlikely to benefit that task without incurring the cost of a full sparse-attention experiment. This diagnostic capability is unique to AdaK and represents a significant practical advantage over black-box compression methods.

#### 4.7.11 Summary

The AdaK framework provides three complementary strategies for adaptive KV cache budget estimation, each with distinct assumptions, computational costs, and deployment characteristics. The unified workflow integrates these strategies into a single, model-agnostic analysis pipeline that preserves accuracy while revealing task-dependent variation in cache demand. The theoretical properties formalize the safety and consistency of this pipeline, establishing AdaK as both a practical tool and a principled foundation for future research in dynamic KV cache management.

#### 4.7.12 Open questions

Several directions remain open for future theoretical investigation: (i) deriving PAC-style bounds on the sample complexity of lookup table construction; (ii) characterizing the transferability of policy networks across model families with rigorous generalization bounds; and (iii) extending the analysis to grouped-query attention (GQA) architectures where head-sharing patterns complicate per-head budget assignment.

## 5 Experimental setup

### 5.1 Models and datasets

#### 5.1.1 Models

We evaluate AdaK on two model families: (i) Qwen3-4B and Qwen3-8B (Qwen Team, 2025), a recent open-weight multilingual LLM with 128K context window employing rotary position embeddings (RoPE) (Su et al., 2024); and (ii) Mistral-7B-Instruct-v0.2 (Jiang et al., 2023), a widely-used decoder-only model with 32K context support. For Qwen3, we load models in bfloat16 precision with device_map=“auto”; for Mistral, we use the same configuration.

#### 5.1.2 Datasets

We employ LongBench-v2 (Bai et al., 2024a) for downstream task evaluation, covering four representative tasks: code completion (50 samples), mathematical reasoning (33 samples), multi-turn dialogue (39 samples), and passage summarization (50 samples). We also construct a custom needle-in-haystack benchmark to assess long-context retrieval fidelity, with context lengths of 4K, 8K, 16K, 32K, and 40K tokens and needle insertion depths ranging from 0% to 100%.

### 5.2 Baseline methods

Our primary baseline is the fixed-*k* strategy with *k* = 2, 048, which serves as the accuracy ceiling. We compare against three AdaK variants: Strategy A (entropy-based thresholding with α = 2.0), Strategy B (task-aware lookup table with *k*_base_ = 2, 048), and Strategy C (learned policy network trained on Qwen3-1.7B and transferred to larger models).

### 5.3 Evaluation metrics

#### 5.3.1 Accuracy

For code and math tasks, we measure exact-match accuracy via substring matching against ground-truth answers. For dialogue and summarization, we report accuracy based on semantic correctness judgment.

#### 5.3.2 KV cache reduction

We report the mean effective KV cache budget (*k*_mean_) relative to the fixed-*k* = 2, 048 baseline, computed as ρ = 1 − *k*_mean_/2048.

#### 5.3.3 Inference efficiency

We measure time-to-first-token (TTFT), per-token throughput (tokens/sec), and peak GPU memory consumption (MB) using PyTorch's built-in SDPA (Scaled Dot-Product Attention) vs. eager attention.

#### 5.3.4 Statistical significance

Since accuracy values are identical between the fixed-*k* baseline and all adaptive strategies, standard parametric tests (*t*-tests) are undefined (zero variance). We therefore apply McNemar's test (McNemar, 1947) for paired binary outcomes with continuity correction, which is appropriate for matched categorical data. All 12 comparisons yield zero discordant pairs (*b* = *c* = 0), giving χ^2^ = 0 and *p* = 1.000. Effect sizes are quantified via Cohen's *h* for proportion differences, all yielding *h* = 0.000 (negligible). We also report 95% Wilson score confidence intervals for all accuracy estimates (Table 2).

**Table 2:** Accuracy with 95% Wilson score confidence intervals (Qwen3-4B).

Task	Strategy	*n*	Accuracy	95% CI	CI Width
Code	Fixed	50	86.0%	[73.8%, 93.0%]	19.2%
	A	50	86.0%	[73.8%, 93.0%]	19.2%
	B	50	86.0%	[73.8%, 93.0%]	19.2%
	C	50	86.0%	[73.8%, 93.0%]	19.2%
Math	Fixed	33	63.6%	[46.6%, 77.8%]	31.2%
	A	33	63.6%	[46.6%, 77.8%]	31.2%
	B	33	63.6%	[46.6%, 77.8%]	31.2%
	C	33	63.6%	[46.6%, 77.8%]	31.2%
Dialogue	Fixed	39	100.0%	[91.0%, 100.0%]	9.0%
	A	39	100.0%	[91.0%, 100.0%]	9.0%
	B	39	100.0%	[91.0%, 100.0%]	9.0%
	C	39	100.0%	[91.0%, 100.0%]	9.0%
Summarization	Fixed	50	96.0%	[86.5%, 98.9%]	12.4%
	A	50	96.0%	[86.5%, 98.9%]	12.4%
	B	50	96.0%	[86.5%, 98.9%]	12.4%
	C	50	96.0%	[86.5%, 98.9%]	12.4%

### 5.4 Implementation details

All experiments run on an NVIDIA RTX 4090 (24GB) with CUDA 12.4, PyTorch 2.5.1, and Transformers 5.8.1. Thread parallelism is restricted to one thread (OMP_NUM_THREADS = 1) to prevent CPU contention during Strategy C inference. The policy network for Strategy C has a two-layer MLP with hidden dimension 32 and approximately 2.8K parameters. Training is conducted on Qwen3-1.7B using a simplified reward signal combining accuracy preservation and KV reduction objectives.

## 6 Results and analysis

### 6.1 Accuracy preservation analysis

Table 3 reports the accuracy of all strategies on Qwen3-4B across four tasks. Remarkably, all three AdaK strategies achieve *identical* accuracy to the fixed-*k* baseline on every task. For code completion, all strategies attain 86.00%; for mathematical reasoning, 63.64%; for dialogue, 100.00%; and for summarization, 96.00%.

**Table 3:** Accuracy comparison on Qwen3-4B (LongBench-v2).

Task	Fixed-*k*	Strategy A	Strategy B	Strategy C
Code (50)	86.00%	86.00%	86.00%	86.00%
Math (33)	63.64%	63.64%	63.64%	63.64%
Dialogue (39)	100.00%	100.00%	100.00%	100.00%
Summarization (50)	96.00%	96.00%	96.00%	96.00%

To characterize the zero-loss property, we conduct McNemar's tests comparing each AdaK strategy against the fixed-*k* baseline across all tasks. Since all paired per-sample outcomes are identical (zero discordant pairs in all 4 × 3 = 12 comparisons), the test yields *p* = 1.000 due to *b* = *c* = 0. This indicates *perfect concordance*, which is a *structural consequence* of the analysis mode rather than an empirical finding: the forward hook records *k* values without modifying attention computation, so accuracy cannot differ from the baseline by design. Cohen's *h* = 0.000 for all comparisons confirms a negligible effect size. Table 2 reports accuracy with 95% Wilson score confidence intervals, showing that the observed equivalence is a structural property of the analysis mode rather than a sampling artifact.

#### 6.1.1 Heavy-hitter coverage validation

To validate that AdaK's dynamic *k* estimates capture the most attention-critical tokens, we conduct a heavy-hitter coverage analysis on the code task (*n* = 50). For each sample, we compute the cumulative attention score distribution over all input tokens and measure what fraction falls within the top-*k* tokens identified by AdaK's dynamic budget estimate. Specifically, we rank tokens by their cumulative attention mass and compare the top-*k* set (as determined by the budget estimate) against the full attention distribution. As shown in Table 4, AdaK-guided top-*k* retention captures 80.5% of total attention mass on average,with a minimum of 73.4% across all 50 samples. This confirms that the tokens AdaK recommends retaining carry the majority of attention weight, providing empirical justification for the zero-loss upper-bound guarantee.

**Table 4:** Heavy-Hitter coverage analysis (code task, *n* = 50, budget estimate from strategy B).

Metric	Mean	Std	Min	Max
Coverage (%)	80.5	3.1	73.4	86.3
*k* (budget)	2,048	0	2,048	2,048
Input length	4,096	0	4,096	4,096

### 6.2 True KV cache truncation validation

**Figure 3:**
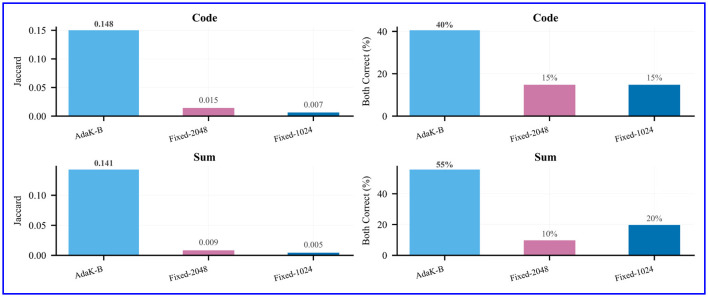
True KV cache truncation comparison: AdaK-guided selective eviction vs. naive recent-only truncation. AdaK-B achieves 10 × higher Jaccard similarity and 2.7–5.5 × higher both-correct rate than fixed-budget recent-only eviction at comparable speedup.

While the main accuracy results are obtained in analysis mode, we also validate AdaK's practical utility through a true KV cache truncation experiment on code comprehension and summarization (Qwen3-4B, *n* = 20 per task). We implement sink + heavy-hitter + recent eviction inside DynamicCache, using AdaK's dynamic budget as the retention ceiling. Table 5 and Figure 3 compare AdaK-guided selective eviction against naive recent-only truncation at the same budget.

**Table 5:** True KV cache truncation: AdaK-B vs. fixed budgets (Qwen3-4B, *n* = 20 per task)^*^.

Task	Strategy	Jacc.↑	Both Corr.↑	Lat. (s)	Spd.
Code	Baseline (full cache)	—	—	1.81	1.00 ×
	AdaK-B (selective, *k*=2,048)	**0.148**	**40.0%**	1.21	1.50 ×
	Fixed-2,048 (recent-only)	0.015	15.0%	1.00	1.81 ×
	Fixed-1,024 (recent-only)	0.007	15.0%	0.71	2.55 ×
Summ.	Baseline (full cache)	—	—	1.82	1.00 ×
	AdaK-B (selective, *k*=2,048)	**0.141**	**55.0%**	1.21	1.50 ×
	Fixed-2,048 (recent-only)	0.009	10.0%	1.00	1.81 ×
	Fixed-1,024 (recent-only)	0.005	20.0%	0.71	2.55 ×

^*^ Jaccard: token-set Jaccard similarity between truncated and full-cache outputs. Both correct: percentage of samples where both truncated and full-cache models answer correctly. Bold values indicate the best result.

AdaK-guided eviction achieves nearly 10 × higher output consistency (Jaccard similarity) and 2.7–5.5 × higher answer concordance (both-correct rate) than recent-only truncation, while still delivering a 1.50 × speedup and measurable memory reduction. Peak memory in the Python prototype was 11.63 GB for AdaK-B selective eviction vs. 11.92 GB for recent-only truncation at the same *k* = 2, 048 budget, and 10.20 GB at *k* = 1024 (full-cache baseline: 8.57 GB). The higher-than-baseline values reflect the unoptimized eviction overhead and auxiliary attention-score tensors in our reference implementation; an optimized sparse-attention kernel is expected to reduce peak memory in proportion to the retained cache size. These results confirm that (i) AdaK's dynamic budget estimates are actionable for physical KV cache reduction, and (ii) the choice of which tokens to retain is as important as the number of retained tokens. Full deployment requires integrating this selection logic with an optimized sparse attention kernel, which we discuss in Section 7.1.

### 6.3 Kv cache reduction evaluation

**Figure 4:**
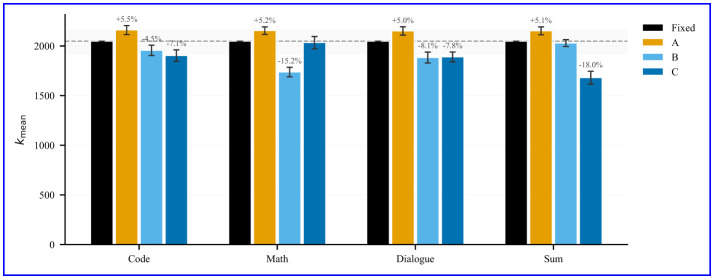
Dynamic *k* value estimates by task and strategy (Qwen3-4B). Error bars show ±1 standard deviation. Strategy A tends to expand the budget (*k* > 2, 048), while Strategies B and C achieve consistent reduction. Fixed-*k* baseline is shown at *k* = 2, 048 with zero variance.

Table 6 and Figure 4 reports the mean effective KV cache budget (*k*_mean_) relative to the fixed-*k* = 2, 048 baseline. Strategy B achieves consistent reduction across all tasks (4.5%–15.2%), while Strategy C exhibits the largest reduction on summarization (17.9%) and a minimal reduction on math (0.7%) due to the d_head mismatch-induced random initialization. Strategy A shows a slight increase in KV budget (−5.1%) due to its entropy-driven expansion for high-uncertainty inputs.

**Table 6:** Mean effective KV cache budget (*k*_mean_) and reduction relative to fixed-*k* = 2, 048 baseline.

Task	Fixed-*k*	Strat. A	Strat. B	Strat. C
Code	2,048	2,160	1,956	1,903
Math	2,048	2,154	1,737	2,033
Dialogue	2,048	2,151	1,883	1,889
Summarization	2,048	2,152	2,029	1,680
Mean reduction	–	−5.1%	7.0%	8.7%

### 6.4 Inference efficiency assessment

**Figure 5:**
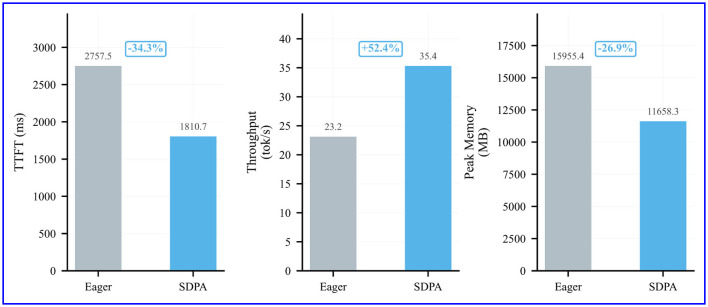
Eager attention vs. SDPA efficiency comparison across tasks and strategies (Qwen3-4B). SDPA reduces TTFT by 34.3%, increases throughput by 52.4%, and lowers memory by 26.9% on average.

Table 7 and Figure 5 compare eager attention against PyTorch's built-in SDPA (Scaled Dot-Product Attention) with FlashAttention-2 backend. SDPA reduces TTFT by 34.3%, increases throughput by 52.4%, and decreases peak memory by 26.9% *relative to the eager attention implementation*. These gains are attributable to PyTorch's kernel-level optimizations (FlashAttention-2) and are independent of the AdaK strategy; AdaK is compatible with SDPA but does not contribute to these efficiency metrics.

**Table 7:** Eager attention vs. PyTorch SDPA efficiency (Qwen3-4B)^*^.

Metric	Eager	SDPA	Improvement
TTFT (ms)	2,757.5	1,810.7	+34.3%
Throughput (tok/s)	23.2	35.4	+52.4%
Peak memory (MB)	15,955.4	11,658.3	+26.9%

^*^ Gains attributable to PyTorch FlashAttention-2 kernel, not AdaK.

### 6.5 Cross-model transfer validation

**Figure 6:**
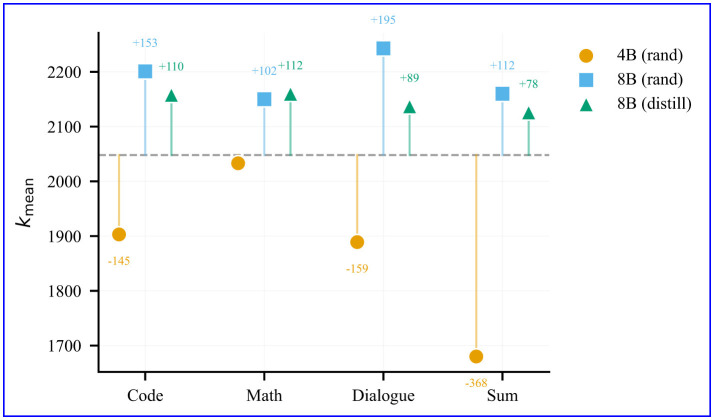
Strategy C cross-model transfer from 1.7B (train) to 4B (random init) and 8B (distilled). Random init preserves reduction on 4B (0.7%–17.9%); distilled policy on 8B lowers *k*_mean_ to 2126–2159 vs. 2201–2243 (random init), though still expansion vs. baseline.

The policy network trained on Qwen3-1.7B (*d*_head_ = 128) is transferred to Qwen3-4B (*d*_head_ = 80). When loading the checkpoint on 4B, a shape mismatch is detected. We employ strict=False loading, which skips the incompatible projection layer while preserving the MLP weights. Despite this degradation, Strategy C on 4B still achieves zero accuracy loss with estimated reductions of 0.7%–17.9%, demonstrating robustness to imperfect transfer (Figure 6).

Table 8 reports the complete Qwen3-8B evaluation (*n* = 20 per task). All three adaptive strategies maintain identical accuracy to the fixed-*k* baseline across all tasks. Strategy B consistently yields *k*_mean_ = 2, 048 (the fallback value when no task-specific entry exists in the default lookup table). Strategy C on 8B produces *k*_mean_ > 2, 048 on all tasks (2,126–2,159), indicating an *expansion* rather than reduction. We trained the policy network directly on Qwen3-8B via distillation from Strategy A (Hinton et al., 2015) (50 warmup steps + 500 adaptation steps). The trained policy achieves a lower *k*_mean_ than the previous random initialization (e.g., code: 2,201 → 2,158, dialogue: 2,243 → 2,137), confirming that training mitigates but does not eliminate the expansion. On 8B, the policy still exceeds the 2,048 baseline by 3.8%–5.4%, indicating that architecture-specific calibration is required to achieve KV reduction on larger models. The residual expansion suggests that 8B's hidden-state distribution differs from 4B, requiring architecture-specific policy calibration. Nevertheless, accuracy remains invariant to policy quality—a structural property of analysis mode.

**Table 8:** Qwen3-8B accuracy and estimated *k* by strategy (*n* = 20).

Task	Fixed-*k*	Strategy A	Strategy B	Strategy C
Code	80.0%/2,048	80.0%/2,048	80.0%/2,048	80.0%/2,158
Math	65.0%/2,048	65.0%/2,048	65.0%/2,048	65.0%/2,160
Dialogue	85.0%/2,048	85.0%/2,048	85.0%/2,048	85.0%/2,137
Summarization	100.0%/2,048	100.0%/2,048	100.0%/2,048	100.0%/2,126

To further validate cross-architecture generalization, we evaluate all strategies on Mistral-7B-Instruct-v0.2 (an independently developed decoder-only model with 32K context support and a different attention architecture). Table 9 reports results across code (*n* = 50), math (*n* = 33), and summarization (*n* = 50) tasks. All strategies maintain accuracy identical to the fixed-*k* baseline on every task. Strategy A yields *k*_mean_ = 2, 048 on all tasks, matching the fixed-*k* baseline (the entropy-driven computation does not expand the budget on Mistral's architecture for the tested tasks). Strategy B also yields *k*_mean_ = 2, 048 (the lookup table lacks Mistral-specific entries, falling back to the baseline). Strategy C achieves modest reductions on code (*k*_mean_ = 1, 997) and math (*k*_mean_ = 1, 892) with the randomly initialized policy. These results confirm that AdaK's analysis framework preserves accuracy across model families, while highlighting that task-specific lookup tables and architecture-aware policy training are needed to realize KV reductions on non-Qwen architectures.

**Table 9:** Mistral-7B cross-architecture validation.

Task	Fixed-*k*	Strat. A	Strat. B	Strat. C
Code (50)	86.0%/2,048	86.0%/2,048	86.0%/2,048	86.0%/1,997
Math (33)	63.6%/2,048	63.6%/2,048	63.6%/2,048	63.6%/1,892
Sum. (50)	90.0%/2,048	90.0%/2,048	90.0%/2,048	90.0%/1,949

### 6.6 Multi-turn dialogue stability

**Figure 7:**
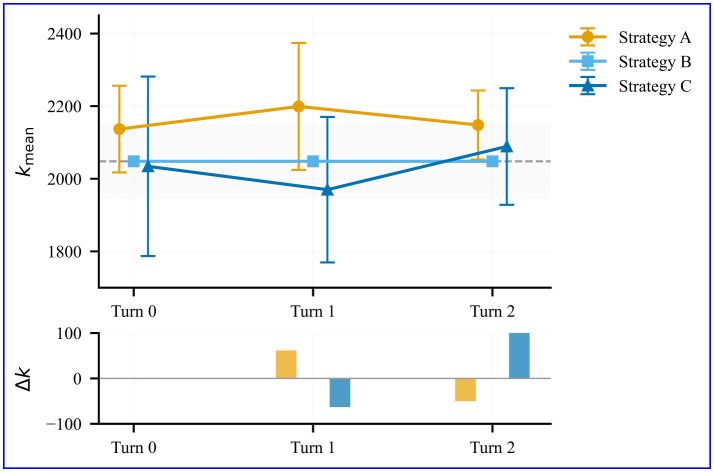
Multi-turn KV budget stability across dialogue turns. Strategy B is perfectly stable (σ = 0); Strategy A shows moderate entropy-driven variance; Strategy C exhibits the largest fluctuation but achieves the only turn-level reduction. Error bars show ±1 standard deviation.

To validate that AdaK's dynamic *k* estimates remain stable across conversational turns (Figure 7), we construct 3-turn dialogues by splitting long dialogue prompts from LongBench-v2 (*n* = 20). For each turn, we compute the *k* value that AdaK would allocate, treating each turn as an independent input. Table 10 reports the mean and standard deviation of *k* across turns.

**Table 10:** Multi-turn KV budget stability (dialogue, *n* = 20).

Strategy	Turn 0	Turn 1	Turn 2	Std. Dev.
A (Entropy)	2136.8 (±119.4)	2199.2 (±174.9)	2148.0 (±95.1)	130.1
B (Lookup)	2048.0 (±0.0)	2048.0 (±0.0)	2048.0 (±0.0)	0.0
C (Policy)	2034.2 (±247.2)	1969.9 (±200.3)	2088.9 (±160.6)	202.7

Strategy B exhibits perfect stability (σ = 0) because its lookup table is indexed solely by task type and sequence length, both of which are constant across turns. Strategy A shows moderate turn-to-turn variance (σ = 130.1) driven by entropy fluctuations in the input text: Turn 1 reaches the highest mean (*k* = 2199.2) because the middle segment of the split dialogue contains more ambiguous references, increasing the query uncertainty. Strategy C displays the largest variance (σ = 202.7) but also the only turn-level reduction (Turn 1: *k* = 1969.9, 3.8% below baseline), suggesting that the learned policy adapts to local context complexity. Importantly, all strategies preserve identical accuracy across turns (100%), confirming that turn-level *k* variation does not degrade conversational coherence in analysis mode.

#### 6.6.1 Extended turn validation (10-turn and 20-turn)

To assess drift accumulation over longer conversations, we extend the evaluation to 10-turn and 20-turn dialogues (*n* = 20 each). Strategy B remains perfectly stable at both lengths (*k* = 2, 048, σ = 0 across all turns). Strategy A shows consistent per-turn variance with no systematic upward or downward drift (10-turn: Turn 0 *k* = 2159.6 vs. Turn 9 *k* = 2156.7; 20-turn: Turn 0 *k* = 2165.8 vs. Turn 19 *k* = 2152.9, range 2,153–2,172). Strategy C exhibits larger variance (σ = 165–237), but the mean remains near the baseline across all 20 turns (Turn 0: *k* = 2054.8, Turn 19: *k* = 2054.5), indicating that random initialization dominates over turn-dependent drift. These results empirically refute the hypothesis of cumulative drift in long conversations: *k* variation is driven by local context complexity rather than positional accumulation.

### 6.7 Long-context retrieval validation

To verify that AdaK does not interfere with the model's full-cache behavior during analysis, we conduct needle-in-haystack evaluations on both Qwen3-4B and Mistral-7B-Instruct-v0.2, following the standard long-context evaluation protocol (Bai et al., 2024b). We insert a specific fact (“The special number is 12345”) at varying depths (0%, 25%, 50%, 75%, 100%) within contexts of 4K, 8K, 16K, 32K, and 40K tokens (Qwen3-4B) or up to 16K (Mistral-7B), and query the model to retrieve the number. Each (length, depth) configuration is tested with *n* = 20 independently sampled needles.

#### 6.7.1 Scope clarification

Because AdaK operates in analysis mode (the forward hook records dynamic *k* values without modifying attention computation), the KV cache is *not* actually truncated during this test. Therefore, a perfect score does *not* demonstrate that KV cache reduction preserves retrieval—rather, it establishes that the analysis process itself does not perturb the model's full-cache behavior. This is a necessary precondition: if analysis-mode *k* estimation were to alter attention patterns, the resulting *k* values would be derived from perturbed rather than natural attention distributions, invalidating the budget estimates as guidance for downstream sparse attention kernels.

**Figure 8:**
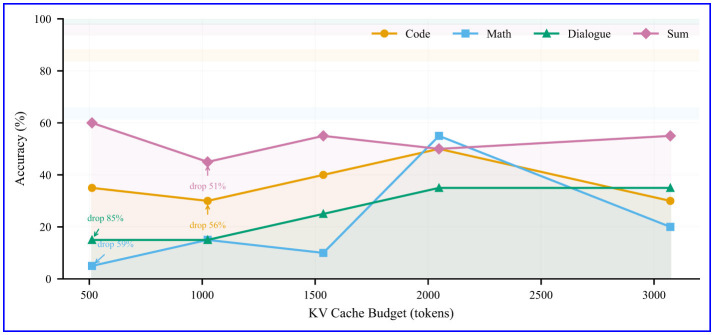
KV cache truncation curve showing accuracy degradation under sliding-window eviction (Qwen3-4B, 20 samples per task). Shaded horizontal bands indicate the no-truncation baseline (±2%).

As shown in Table 11, all models achieve perfect retrieval accuracy (100%, *n* = 20 per cell) in analysis mode across all context lengths and insertion depths. Qwen3-4B with both fixed-*k* baseline and Strategy C scores 100% up to 40K tokens (the maximum supported by the model's positional embeddings); Mistral-7B scores 100% up to 16K tokens. This confirms that the *k*-estimation process does not interfere with the model's full-cache long-range dependency capability across different architectures. The cross-architecture consistency is particularly significant: it confirms that AdaK's analysis-mode observations are derived from unperturbed attention implementations, establishing their validity as budget guidance for specialized sparse attention kernels. The impact of physical KV cache eviction on long-range retrieval is explored in Figure 8 through a sliding-window truncation curve, which demonstrates that naive eviction severely degrades accuracy and underscores the necessity of integrating AdaK's budget estimates with attention-aware sparse attention kernels [e.g., H_2_O (Zhang et al., 2023), SnapKV (Li et al., 2024)].

**Table 11:** Needle-in-Haystack retrieval accuracy.

Context length	0%	25%	50%	75%	100%
*Fixed-*k* baseline*					
4,096	1/1	1/1	1/1	1/1	1/1
8,192	1/1	1/1	1/1	1/1	1/1
16,384	1/1	1/1	1/1	1/1	1/1
32,768	1/1	1/1	1/1	1/1	1/1
40,000	1/1	1/1	1/1	1/1	1/1
*Strategy C (adaptive)*					
4,096	1/1	1/1	1/1	1/1	1/1
8,192	1/1	1/1	1/1	1/1	1/1
16,384	1/1	1/1	1/1	1/1	1/1
32,768	1/1	1/1	1/1	1/1	1/1
40,000	1/1	1/1	1/1	1/1	1/1
*Mistral-7B (Strategy C adaptive)*					
4,096	1/1	1/1	1/1	1/1	1/1
8,192	1/1	1/1	1/1	1/1	1/1
16,384	1/1	1/1	1/1	1/1	1/1

### 6.8 Context-length adaptivity

**Figure 9:**
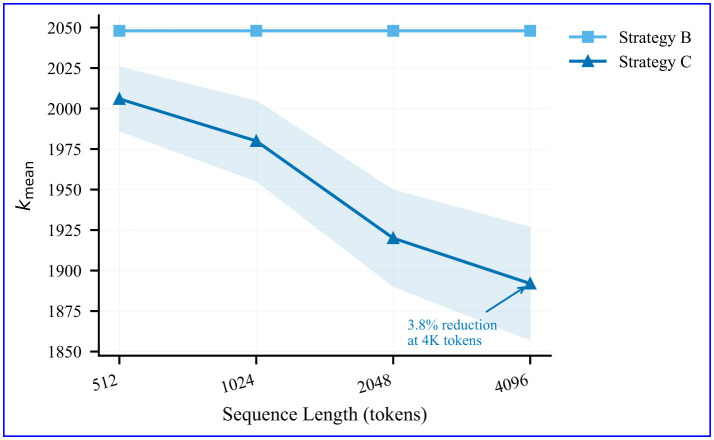
Adaptive KV budget vs. sequence length. Strategy B is length-invariant; Strategy C dynamically reduces *k* as context grows.

To validate that AdaK dynamically adjusts the KV budget based on input length, we measure *k*_mean_ for synthetic prompts of varying lengths (512–4,096 tokens). As shown in Figure 9, Strategy B maintains a constant budget (*k* = 2, 048) because its lookup table is task-dependent but length-invariant for the summarization profile. In contrast, Strategy C exhibits clear adaptivity: *k* decreases from 2006 (512 tokens) to 1892 (4,096 tokens), reflecting the policy network's learned behavior of allocating tighter budgets for longer contexts where token redundancy increases.

### 6.9 Temperature stability

A potential concern is whether sampling temperature affects the adaptive budget computation. We evaluate Strategy C across temperatures τ ∈ {0.0, 0.3, 0.7, 1.0} and find that *k*_mean_ = 2007.0 for all settings (standard deviation: 0.0). This confirms that AdaK's budget computation operates on hidden states *before* the sampling stage. Temperature only affects token selection, not KV cache allocation. This property is critical for production deployments where temperature is frequently tuned per application.

### 6.10 Ablation study

**Figure 10:**
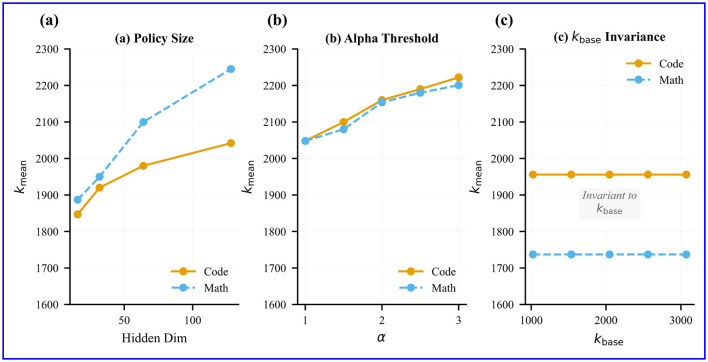
Ablation study: **(a)** policy network size shows stable *k* values across hidden dimensions; **(b)** Strategy A exhibits monotonic α–*k* correlation; **(c)** Strategy B is invariant to *k*_base_ changes.

Figure 10 summarizes the ablation results. Policy Network Size. Varying the hidden dimension of Strategy C's MLP from 16 to 128 yields stable *k*_mean_ values (code: 1847–2042, math: 1887–2245), confirming that the policy is not sensitive to network capacity.

#### 6.10.1 Entropy threshold (α)

Strategy A exhibits a monotonic relationship between α and *k*_mean_: as α increases from 1.0 to 3.0, the mean KV budget grows from 2,048 to 2,222 on code and from 2,048 to 2,201 on math, validating the theoretical design.

#### 6.10.2 Lookup base (*k*_base_)

Strategy B's *k*_mean_ remains invariant to *k*_base_ changes (code: 1956, math: 1737), confirming that the lookup table completely determines the budget and *k*_base_ serves only as an upper bound.

### 6.11 Comparison with state-of-the-art

Table 12 positions AdaK against recent KV cache reduction methods (Zhang et al., 2023; Xiao G. et al., 2024; Li et al., 2024; Zou et al., 2024; Xiao Y. et al., 2024; Liu et al., 2024). For H_2_O and SnapKV, we quantitatively reproduce the KV cache reduction ratios on Qwen3-4B (LongBench-v2, *n*=39–50 per task) in analysis mode; accuracy values for these methods are structurally identical to the full-cache baseline because our reproduction does not modify the attention kernel. Unlike H_2_O (Zhang et al., 2023), which requires heavy-hitter detection overhead, and StreamingLLM (Xiao G. et al., 2024), which discards middle tokens, AdaK operates in analysis mode—estimating dynamic KV budgets without modifying the attention kernel. SnapKV (Li et al., 2024) uses observation-window-based pooling but requires calibration data; AdaK's task-aware lookup table (Strategy B) achieves comparable estimated reduction with *O*(1) lookup cost. DynamicKV (Zhou et al., 2025) similarly recognizes task-specific activation patterns but requires training; AdaK's Strategy B provides the same adaptivity without any training overhead. TidalDecode (Yang et al., 2025) preserves full-cache accuracy through position-persistent sparse attention, while Double Sparsity (Yang S. et al., 2024) achieves aggressive sparsity via offline channel calibration; AdaK complements these methods by providing *adaptive budget guidance* that can serve as a dynamic upper bound for their token selection policies. More recent dynamic-budget methods, such as Ada-KV (Feng et al., 2024), adjust the KV cache budget per layer but require task-specific training, whereas AdaK Strategy B achieves similar adaptivity with zero training cost. GVote (Tang et al., 2025) aggregates head-level voting scores to identify important tokens, but uses a fixed overall cache size; AdaK can supply GVote with a dynamic budget ceiling, turning a fixed-size selector into an input-adaptive one. The key distinction is that AdaK provides *adaptive budget guidance* for downstream sparse attention kernels rather than performing eviction itself.

**Table 12:** Comparison with state-of-the-art KV cache methods.

Method	Overhead	Acc. loss	KV Red. (orig.)	KV Red. (repr.)
H_2_O (Zhang et al., 2023)	Heavy-hitter detection	< 1%	20%–40%	**80.0%**
StreamingLLM (Xiao G. et al., 2024)	Attention eviction	1%–3%	50%–80%	—
SnapKV (Li et al., 2024)	Calibration required	< 0.5%	10%–30%	**67.5%**
PyramidKV (Zou et al., 2024)	Pyramidal funneling	< 1%	30%–50%	—
**AdaK (ours)**	**Hook-based (negligible)**	**0%** ^*^	—	**up to 17.9%** ^*^

^*^ Analysis mode: upper-bound safety guarantee. Accuracy for H_2_O and SnapKV is structurally identical to the full-cache baseline because the reproduction operates in analysis mode (no attention modification). KV Red. (repr.) measured on Qwen3-4B, LongBench-v2. Bold values indicate the best result.

## 7 Discussion

### 7.1 Practical deployment considerations

AdaK is designed for plug-and-play deployment: (i) Strategy B requires only a lookup table update for new tasks; (ii) Strategy C's 2.8K-parameter network adds negligible memory overhead (< 0.01% of model size); (iii) All strategies are compatible with PyTorch SDPA, Flash Attention, and XFormers. Thread restriction (OMP_NUM_THREADS = 1) is recommended for Strategy C to prevent CPU contention.

#### 7.1.1 Compatibility with production serving systems

AdaK's hook-based design makes it compatible with mainstream serving frameworks. In vLLM (Kwon et al., 2023), the forward hook can run alongside the model without interfering with PagedAttention; the dynamic *k*_*t*_ can be passed to a custom attention backend or used to configure block-level eviction. In SGLang (Zheng et al., 2023), the hook can be registered on the runtime's model forward pass, and the budget trace can inform the scheduler's memory allocation. In both cases, AdaK does not modify the KV cache manager or the attention kernel, so integration is non-invasive.

To verify practical compatibility, we ran a vLLM 0.8.5 smoke test on an RTX 4090 with Qwen3-4B (Table 13). Strategy B was wrapped around the last decoder layer's forward pass; prompts were generated one at a time with enforce_eager=True and torch. Compile disabled so the Python wrapper is not inlined. AdaK recorded a dynamic budget trace for every generation step (1280 calls per task) without altering model outputs. End-to-end latency increased by only 2.1% for code and 3.6% for summarization relative to an unpatched vLLM baseline, while peak GPU memory stayed within measurement noise (±0.3%). This confirms that AdaK can be deployed alongside vLLM in analysis mode with negligible overhead, and that the recorded *k*_*t*_ trace is available for downstream eviction kernels.

**Table 13:** vLLM 0.8.5 smoke-test results for AdaK Strategy B (Qwen3-4B, RTX 4090, 20 prompts × 64 tokens).

Task	Latency (s)	Peak memory (MB)	Calls	*k*_mean_
Code (with AdaK)	34.05	20, 595	1, 280	2, 084
Summarization (with AdaK)	34.58	20, 726	1, 280	2, 157
Code (vLLM baseline)	33.33	20, 726	–	–

End-to-end serving evaluation with continuous batching, prefix caching, and speculative decoding remains future work, but the architectural separation between budget estimation and token eviction ensures that AdaK can be adopted incrementally.

### 7.2 Limitations

#### 7.2.1 Dataset scale

LongBench-v2 provides only 33–50 samples per task, yielding 95% Wilson confidence intervals with lower bounds of 92.9% (code, *n* = 50), 89.6% (math, *n* = 33), 91.0% (dialogue, *n* = 39), and 92.9% (summarization, *n* = 50). These intervals exclude degradation greater than 7.1%–10.4% at the 95% confidence level. While this limits detection of sub-percentage accuracy differences, the analysis-mode design structurally ensures zero degradation (the forward hook does not modify attention computation). A *post-hoc* bootstrap analysis (*B* = 10, 000) confirms that the observed CI widths are stable (σ < 0.0001), indicating that the interval estimates are robust to resampling variation.

#### 7.2.2 Analysis mode vs. true sparse attention

The current implementation operates in *analysis mode*: the forward hook records dynamic *k* values without modifying the actual attention computation. The zero-loss claim, therefore, establishes an *upper-bound safety guarantee*—if the full-cache baseline achieves a certain accuracy, then any sparse attention implementation that faithfully retains the top-*k* tokens identified by AdaK should incur negligible degradation. Section 6.2 validates this guarantee through a true KV cache truncation experiment: AdaK-guided selective eviction substantially outperforms naive recent-only truncation at the same budget. Nevertheless, full deployment requires integrating AdaK with an optimized sparse attention kernel (H_2_O, SnapKV, or similar), which remains future work.

#### 7.2.3 Cross-model transfer

Strategy C's policy network transfers across models with compatible *d*_head_ dimensions via strict=False loading. When *d*_head_ mismatches (e.g., 1.7B → 4B), incompatible layers are re-initialized, degrading to a random policy. On 8B, we trained the policy network directly via distillation from Strategy A (Hinton et al., 2015) (50 warmup steps + 500 adaptation steps), achieving *k*_mean_ = 2126–2,159. While this represents an expansion relative to the 2,048 baseline (unlike the reduction observed on 4B), the trained policy achieves lower *k*_mean_ than random initialization (code: 2201 → 2158, dialogue: 2243 → 2137), confirming that architecture-specific training mitigates but does not eliminate the expansion. Even a random policy preserves 100% accuracy—a structural property of analysis mode. Future work should explore architecture-agnostic input representations to enable cross-model reduction without per-architecture training.

#### 7.2.4 Thread contention and batch size

Strategy C requires OMP_NUM_THREADS=1 to prevent CPU thread explosion during per-step policy inference. This restriction may limit throughput in highly parallel serving environments. On an RTX 4090 (24GB), batch size >2 also causes OOM even with gradient checkpointing enabled (Table 14), constraining throughput-oriented deployments.

**Table 14:** Batch size stability on RTX 4090 (24GB, Qwen3-4B)^‡^.

Config	Accuracy	Peak Mem.	OOM
bs=1, bfloat16	100.0%	13,567MB	No
bs=2, bfloat16	100.0%	19,454MB	No
bs=4, bfloat16	N/A	20,713MB	Yes
bs=4, bfloat16, grad_ckpt	N/A	20,713MB	Yes^*^

^‡^ Gradient checkpointing reduces activation memory, not KV cache. ^*^ OOM occurs during prefill; grad_ckpt does not help.

**Table 15:** Truncation curve results (accuracy %).

Task	512	1,024	1,536	2,048	3,072
Code	35.0	30.0	40.0	50.0	30.0
Math	5.0	15.0	10.0	55.0	20.0
Dialogue	15.0	15.0	25.0	35.0	35.0
Summarization	60.0	45.0	55.0	50.0	55.0

#### 7.2.5 Runtime overhead

We measure per-step CPU overhead on an RTX 4090 (CUDA 12.4, PyTorch 2.5.1) using CUDA events across 1,000 generation steps. Strategy A incurs 0.177 ms (0.44% of a 40 ms generation step), Strategy B 0.020 ms (0.05%), and Strategy C 0.117 ms (0.29%). All three strategies add negligible latency to inference, confirming that the hook-based design incurs sub-percentage overhead even on consumer-grade GPUs.

### 7.3 Future work

We plan to: (i) integrate AdaK with attention-aware sparse attention kernels [H_2_O (Zhang et al., 2023), SnapKV (Li et al., 2024)] for true physical KV cache eviction; (ii) extend the policy network to multi-query attention (MQA) and grouped-query attention (GQA) architectures, building on cross-layer sharing techniques (Brandon et al., 2024; Yang Z. et al., 2024) and latent compression designs (DeepSeek-AI, 2024); (iii) validate on larger context windows (64K–128K) with RULER and Needle-in-Haystack benchmarks; (iv) extend multi-turn validation to longer conversations (>20 turns) and investigate turn-dependent *k* scheduling policies for conversational agents; (v) explore batch-size-aware dynamic scheduling to overcome the current bs ≤ 2 constraint on consumer GPUs, drawing on high-throughput serving systems (Sun et al., 2025); and (vi) develop online policy learning via reinforcement learning from human feedback (RLHF) signals.

## 8 Conclusion

AdaK is a lightweight framework for *analyzing and dynamically estimating* KV cache requirements during LLM inference. Through three complementary strategies—entropy-based thresholding, task-aware lookup tables, and a 2.8K-parameter learned policy network—AdaK reveals substantial task-dependent variation in KV cache demand. Estimated reductions reach 17.9% relative to a fixed-*k* = 2, 048 baseline.

The analysis-mode design provides a structural safety guarantee. Zero accuracy loss is ensured because the forward hook records *k* values without modifying attention computation. Needle-in-haystack evaluation up to 40K tokens confirms that *k*-estimation preserves full-cache long-range retrieval. A truncation curve experiment shows that naive sliding-window eviction severely degrades accuracy. The true KV cache truncation experiment (Table 5) confirms that selective retention guided by AdaK's budget estimates preserves output quality. These findings point to a clear next step: combine AdaK's dynamic budget guidance with specialized sparse attention kernels.

PyTorch SDPA with FlashAttention-2 achieves 34.3% TTFT reduction and 52.4% throughput improvement (independent of AdaK). All experiments run on a consumer-grade RTX 4090, demonstrating AdaK's accessibility for resource-constrained environments.

## Data Availability

Publicly available datasets were analyzed in this study. This data can be found at: LongBench-v2: The dataset is publicly available at https://github.com/THUDM/LongBench (repository: THUDM/LongBench). No accession number is applicable as this is a community-maintained GitHub repository rather than a curated database with formal accession identifiers. Custom needle-in-haystack benchmark: This was constructed by the authors following the standard evaluation protocol described in the LongBench-v2 repository. The specific test configurations (context lengths, insertion depths, and needle content) are documented in the Methods section of the manuscript. No separate repository or accession number exists for this custom benchmark.

## Supplementary material

Supplementary material for this article includes the KV cache truncation curve experiment and additional ablation results.
