# Supplementary material for: AdaK: adaptive KV cache budget estimation framework for analyzing long-context large language model inference
Source: Front Artif Intell. 2026 Aug 5;9:1892739. doi: 10.3389/frai.2026.1892739 (PMC13486163; doi:10.3389/frai.2026.1892739)
Supplement: Supplementary file 1 [file Data_Sheet_1.pdf]

Supplementary Material for
"AdaK: Adaptive KV Cache Budget Estimation Framework for Analyzing Long-Context Large Language Model Inference"

Tianjun Shao

Overview

This supplementary document provides additional algorithmic details, hyperparameter settings, and reproducibility information for the AdaK framework described in the main manuscript. It is intended to help readers reproduce the analysis-mode experiments and adapt AdaK to their own models.

1 Detailed AdaK Inference Workflow

Algorithm 1 presents the full analysis-mode inference workflow. The hook registers on the last decoder layer and records a dynamic budget k_t at every generation step without modifying the attention computation.

2 Hyperparameters

Table 1 lists the default hyperparameters used for the main experiments. All experiments use full-precision $bfloat16$ model weights and greedy decoding (temperature $= 0$).

Table 1: Default hyperparameters for AdaK experiments.

**Parameter**	**Description**	**Value**
k_{base}	Baseline KV cache budget	2048
k_{min}	Minimum allowed budget	512
k_{max}	Maximum allowed budget	full context length
α	Entropy scaling exponent (Strategy A)	2.0
ϵ	Numerical stability term	10^{-9}
H_{norm}	Entropy normalization factor	$\log(k_{base})$
Learning rate	Strategy C policy training	10^{-3}
Training steps	Strategy C policy training	500
Batch size	Strategy C policy training	1
Optimizer	Strategy C policy training	Adam
Warmup steps	Strategy C distillation	50

3 Task-Aware Lookup Table (Strategy B)

Table 2 shows the lookup table constructed on the validation split of LongBench-v2 for Qwen3-4B. The table maps a (task, sequence length) pair to a target KV cache budget k^*.

Algorithm 1 AdaK Analysis-Mode Inference

Require: Model $\mathcal{M}$, Tokenizer $\mathcal{T}$, Input text x
Require: Strategy $s \in \{A, B, C\}$, Parameter set θ
Ensure: Generated text y, Dynamic budget trace $\{k_t\}_{t=1}^{T}$

$tokens \leftarrow \mathcal{T}$.encode($x$)
$KV_{cache} \leftarrow$ init_cache()
$k\_history \leftarrow []$
Register forward hook $\mathcal{H}$ on the last attention layer of $\mathcal{M}$
while not eos **do**
  $out \leftarrow \mathcal{M}$.generate_step($tokens, KV_{cache}$)
  $q \leftarrow \mathcal{H}$.query {Current-step query vector}
  if $s = A$ **then**
    $scores \leftarrow \text{softmax}(qK^T/\sqrt{d_{head}})$
    $H \leftarrow -\sum scores \cdot \log(scores + \epsilon)$
    $k^* \leftarrow k_{base} \cdot \alpha^H$
  else if $s = B$ **then**
    $task \leftarrow$ detect_task(x)
    $k^* \leftarrow \text{LookupTable}[task, \text{len}(tokens)]$
  else if $s = C$ **then**
    $q_{per\_head} \leftarrow q.\text{reshape}(n_{heads}, d_{head})$
    $\gamma \leftarrow \text{PolicyNetwork}(q_{per\_head})$
    $k^* \leftarrow k_{base} \cdot \text{sigmoid}(\gamma)$
  end if
  $k^* \leftarrow \text{clip}(k^*, k_{min}, k_{max})$
  $k\_history$.append(k^*)
  $token_{next} \leftarrow \text{argmax}(out.\text{logits})$
  $tokens \leftarrow \text{concat}(tokens, token_{next})$
  KV_{cache}.update($out.k, out.v$)
end while
Remove forward hook $\mathcal{H}$
$y \leftarrow \mathcal{T}$.decode($tokens$)
return $y, k\_history$

When a test sample does not match any table entry, Strategy B falls back to k_{max} (full cache) to preserve the zero-loss guarantee.

4 vLLM Smoke-Test Configuration

The vLLM 0.8.5 smoke test described in Sec. 7.1 of the main manuscript uses the following configuration:

- vLLM version: 0.8.5
- Model: Qwen3-4B-Instruct
- GPU: NVIDIA RTX 4090 (24 GB)
- Batch size: 1 (sequential generation)
- `enforce_eager=True`
- torch.compile disabled

Table 2: Example Strategy B lookup table for Qwen3-4B on LongBench-v2.

Task	Mean length	k^*
Code completion	12,800	1942
Dialogue	11,200	2157
Summarization	14,500	2084
Math reasoning	8,900	2032
Question answering	10,100	1986

- Precision: `bfloat16`
- Max output length: 64 tokens
- Number of prompts: 20 per task
- Hook layer: last decoder layer

The AdaK hook is registered on the model's forward pass inside vLLM before the generation loop begins. At each decoding step, the hook extracts the query vector from the last attention layer, computes k_t using Strategy B, and appends it to a trace buffer. The recorded trace is flushed to disk after generation completes.

5 Model and Dataset Details

5.1 Models

- **Qwen3-4B-Instruct** and **Qwen3-8B-Instruct**: loaded in `bfloat16` with `device_map="auto"`.
- **Mistral-7B-Instruct-v0.2**: loaded in `bfloat16` with 32K context support.

5.2 Datasets

All long-context evaluation uses the LongBench-v2 benchmark. Per-task sample sizes range from 33 to 50, following the official benchmark release.

Data and Code Availability

The datasets analyzed for this study are publicly available in the LongBench-v2 benchmark (`https://github.com/THUDM/LongBench`). Algorithmic details, hyperparameters, and configuration information required to reproduce the analysis-mode experiments are provided in this supplementary document and in the main manuscript. Full implementation code is being prepared for release as part of ongoing follow-up work.
